# Assessing the level of digitalization and robotization in the enterprises of the European Union Member States

**DOI:** 10.1371/journal.pone.0254993

**Published:** 2021-07-22

**Authors:** Jarosław Brodny, Magdalena Tutak

**Affiliations:** Silesian University of Technology, Gliwice, Poland; Gonbad Kavous University, ISLAMIC REPUBLIC OF IRAN

## Abstract

One of the main reasons for the dynamic global economic development observed in recent years is the process of digitalization, referred to as Industry 4.0. The significance of digitalization for this development is appreciated by the EU-27. In order for these actions to be effective, it is necessary to diagnose the current level of digitalization in the EU-27countries. The article presents the results of the assessment of the level of digitalization of enterprises in the EU-27 countries. An empirical analysis was conducted using 16 determinants which describe the digitalization in a sample of 27 EU countries. Based on the adopted criteria and the Technique for Order Preference by Similarity to an Ideal Solution method, these countries were divided into four classes in terms of the level of digitalization. The analysis looked at the size of enterprises and was performed independently for small, medium and large enterprises. The adopted indicators allowed for the analysis of similarity between the EU-27 countries in terms of digitalization, using the Kohonen’s networks. The result of this research was the division of the EU-27 countries into groups, also taking into account the size of studied enterprises. Due to the immensely diverse EU-27 economy, such a huge undertaking as the digital transformation process requires building logical internal "digital coalitions". The designated assessment and similarity between countries creates such opportunities, also in terms of building an effective policy to support these processes by the EU. This increases the chances of success of joint ventures and building a sustainable European community based on the latest technologies.

## 1. Introduction

The digital transformation of the global economy is closely linked to the introduction of new technologies and is referred to as Industry 4.0 [1–3]. This process is currently gaining momentum, and the pandemic caused by the SARSCovid-19 coronavirus has accelerated it even further. It turns out that companies with a high degree of digitalization, automation and robotization of their production and service processes are less affected by the pandemic than those based on the traditional forms of their implementation [4]. Their flexibility and ability to respond quickly to changes, being less dependent on the human factor, has made these companies more competitive. The reason for this is the introduction of digital technologies and other innovative solutions from the Industry 4.0 area [5].

It is obvious that the basis for the development of the digital economy is the implementation of advanced cyber-physical systems, based on the analysis of large data sets on production processes in order to optimize them. These activities, combined with the robotization and automation of production processes, result in the support and, in many cases, replacement of human work by machines [6,7]. The digitalization, automation and robotization of production processes have and will have an increasing impact on the structure of employment [8–11].

Nevertheless, it is obvious that the dynamic changes in the digitalization and robotization of production processes are mainly due to the development of data transmission and processing technologies, which, in conjunction with the development of technical means, creates exceptionally favorable conditions for building a digital economy. In these processes, large data sets from mobile and ubiquitous Internet connectivity, cloud computing, big data analytics, artificial intelligence, 3D printing, and many other solutions, are being used on an increasing scale [12].

The digital transformation process has become a key tool to improve the efficiency and competitiveness of companies, which are constantly looking for innovative solutions to optimize their production. However, in order to achieve this goal, it is also essential to apply new business models and product definitions, as well as changes in the way they communicate with customers [13]. The digital transformation of enterprises and entire economies associated with the idea of Industry 4.0 concerns many areas of their activities and affects all their resources [14–16].

Digital transformation creates great opportunities for the economic development of individual countries and entire regions, as well as improving the quality of life of their residents. However, its effects (apparently both positive and negative) will be experienced in virtually all areas of economic, social and political life [17–19]. The most important technologies related to digital transformation, which find wide practical application, are mainly communication technologies (Internet, wireless and mobile networks, etc.), data collection (sensorics, recognition of speech, writing, face and other images) as well as processing and analyzing these data (machine learning, neural networks, artificial intelligence algorithms) [20–22].

From the perspective of manufacturing enterprises, the use of digital solutions is particularly evident in the introduction of new technologies, which results in the robotization and automation of production processes, among other aspects. Currently, the robotization and automation of production processes is considered very beneficial for enterprises. By improving quality, productivity, and efficiency of these processes, including safety, competitive advantage can be more easily achieved. Of great importance is also the support and replacement of human labor in places where there is a threat to their health or life. The interconnection of machines and various types of equipment as well as efficient data transmission enable the implementation of multiple tasks, which through higher productivity and quality as well as reliability can improve efficiency. This makes companies become interested in introducing new digital solutions [23,24].

In addition, the governments of individual countries realize that investments in the digital economy are fully justified and that without the development of this industry they will not be able to ensure the satisfactory pace of development of their countries. At the level of the EU, which developed the Digital Single Market Strategy in 2015 [25], the European Commission is working on identifying and collecting data to assess the social and economic processes associated with digital transformation. The importance of the issue of digitalization and robotization of enterprises is also evidenced by its inclusion in the political strategy for 2019–2024.

In 2021, the Digital Europe Program with a budget of 8.1 billion euros began in the EU. Its aim is to financially support the digital transformation of European societies and economies. As part of the EU budget for 2021–2027, the program is to provide funding for projects in five key areas: supercomputing, artificial intelligence, cyber security, advanced digital skills and the use of digital technologies in the economy [26]. Digitalization is also intended to be the pillar of the EU’s economic recovery from the Covid-19 pandemic, with particular emphasis on small and medium-sized enterprises. Approximately 20% of the Recovery and Resilience Facility is envisaged for this purpose.

All these actions undertaken by the EU show how much attention is paid to the process of digitalization of the economy and the development of the Industry 4.0 idea. This is a difficult process as the EU consists of 27 countries with large differences in economic development, the wealth of their societies and different levels of technical saturation of the economy. Also, in terms of innovation, individual EU countries are extremely different. However, the process of digital transformation requires huge financial resources, for which many companies do not have adequate means. Moreover, most of the EU countries, on their own, are not able to provide adequate support for these processes. Therefore, it is essential to direct the EU financial policy in such a way that as many countries and companies as possible can benefit from the funds allocated for the construction of digital Europe. That is why this funding must be preceded by a thorough study of the current state of digitalization and robotization processes in individual countries. It is also advisable to determine the degree of this advancement from the point of view of the size of enterprises.

The existing publications on the digitalization and robotization as well as automation of production processes in enterprises provide a lot of scientific information related to them [27–61]. However, they fail to refer to the comprehensive assessment of the degree of the digitalization and robotization of the EU enterprises based on their size and similarities between these countries. The lack of such analysis limits the possibility of a comprehensive approach to the assessment of these processes and a more global view of the EU economy in the context of the ongoing digital transformation.

The analysis of the presented problem prompted the authors to formulate the following three research questions, which clarify the subject of the research, order the conducted analysis and enable the evaluation of the results and formulation of future research directions.

1. What is the level of digitalization and robotization across the EU countries?
2. What is the level of digitalization and robotization among enterprises in the EU countries according to their size?
3. What is the similarity between the EU countries in terms of the digitalization and robotization of enterprises of these countries, taking into account the size of these enterprises?

It is reasonable to state that the work presents a new and original approach to the evaluation of the process of the digitalization and robotization of the EU countries and the analysis of their similarity in this regard.

The first factor proving the originality of the presented work is the selection of a set of as many as 16 indicators (determinants) characterizing the process of the digitalization and robotization of enterprises in the most relevant areas for this process. Such a broad and comprehensive approach to studying this issue has not been done before. The second original factor of the work is the inclusion of the size of companies implementing digital technologies in the studied countries. The analysis was conducted for all enterprises in general and additionally for small-, medium- and large-sized businesses. This makes it possible to allocate financial resources to the appropriate groups of enterprises and encourage them to cooperate both nationally and internationally. The third factor concerns the use of the Technique for Order Preference by Similarity to an Ideal Solution (TOPSIS) method to assess the level of the digitalization and robotization of the EU enterprises. It is one of the most popular methods used for multi-criteria analysis of various types of problems, including economic ones. Another factor proving the originality of this work is the similarity analysis between the EU countries in terms of the level of digitalization and robotization of enterprises. The Kohonen’s artificial neural networks were used for these analyses, and their result was the designation of similar groups of the EU countries. The designation of these groups creates great opportunities for cooperation between countries in these groups. This concerns mainly: applying for EU funds, implementation of common digital policy, cooperation between companies, building the 4.0 society, system integration, cyber security and many other areas related to the process of digitalization and robotization.

## 2. Literature review

### 2.1. Key terms related to Industry 4.0

The global dynamic economic development and the associated process of creating more and more modern and intelligent factories based on new technologies are closely related to the concept of Industry 4.0. These changes are linked to many concepts that describe the occurring processes, developed technologies and other elements associated with them. From the point of view of this article, the terms Industry 4.0, digitalization, automation and robotization, big data and cloud computing are particularly important.

Industry 4.0 is a concept that involves the process of technological and organizational transformation of companies, as well as social changes resulting from the development and implementation of new technologies (Internet, 3D printing, etc.). This transformation includes, for example, value chain integration, introduction of new business models and digitalization of products and services [27–37].

According to Gartner’s IT Glossary, digitization is the process of changing from analog to digital form [62]. Digitalization, in turn, is the use of digital technologies to change a business model and provide new revenue and value-producing opportunities. It is the process of moving to a digital busines [63].

Robotization means replacing human labor with devices (machines/robots) that are programmed and controlled by them. The scope of work and activities that robots perform, replacing humans, is very diverse [64]. On the other hand, automation is the process of supporting or replacing repetitive activities, performed by humans, in an automatic way by devices and machines [65]. In this process, there is an increase in the share of machines in production processes by reducing the share of human labor.

In relation to robotization, which involves the modification of activities, automation interferes with application software to perform certain operations (or parts of them) by itself.

Recently, the term Big Data, which defines processes related to the acquisition, retrieval, collection and processing of large data sets, has also become significant. The main goal of these processes is to extract new information and knowledge, and in a further stage, also wisdom from these datasets.

The concept of Cloud Computing is closely related to Big Data. It defines a form of computing service available over the Internet. A number of processes involving the so-called cloud computing are associated with this concept. Currently, a form of access to remote computing tools has been increasingly popular among enterprises and other users, also providing the possibility of data storage [66].

### 2.2. Theoretical background

The Industry 4.0 concept was launched in Germany in 2011 as part of a high-tech strategy to address new economic challenges and ensure the competitiveness of the German production industry [27].

Undoubtedly, the direction of development initiated by these changes will be dominant in the global economy for the next few years. The World Economic Forum estimates that the total global value of the digital transformation process will have exceeded $100 trillion by 2025 [28]. This demonstrates the global nature and irreversibility of this process. The effects have been and will be the increasing changes in production processes, the labor market, consumption and the functioning of state institutions and social life. They result primarily from the need to adapt to the growing economic competition through the use of new innovative technologies, and the launch of new, more and more technologically advanced products on the market.

In the case of the fourth industrial revolution (Industry 4.0), Rüßmann et al. [29] and Wee et al. [30] identified nine technology pillars that have a significant impact on the global economy. These pillars include Big Data Analytics [31], Optimization and Simulation, Cloud Technologies [32], Virtual and Augmented Reality (VR/AR) [33], Horizontal and Vertical Integration of Systems, Industrial Internet of Things (IIoT) [34], Additive Manufacturing Technologies (3D printing) [35], Autonomous Robots, and Cyber Security [36]. These pillars identify the main areas of change associated with the Industry 4.0 concept and are commonly used to promote it.

Therefore, it can be assumed that the basis of the Industry 4.0 concept is the integration of different systems, through the use of digital resources, to build different types of systems, including intelligent systems that, by communicating with each other, can make certain decisions and perform various operations with much less human involvement than before [37].

It is clear that the digitalization of production and business processes through the implementation of smarter machines and equipment can bring many economic and business benefits. In a free market, with the free movement of capital, knowledge and people, it leads to the increased competitiveness, productivity and efficiency of production processes. It also creates opportunities for the efficient use of resources, and thus waste reduction [38,39]. For the implementation of the principles of the circular economy, and the economy of sustainable development, the concepts like digitalization, automation and robotization of production processes can have immensely positive effects. Through the full control of these processes, it creates the possibility of significant reduction of their adverse impact on the environment, among other advantages.

Changes associated with the digitalization of the economy also lead to radical changes in communication processes and various types of internal and external relations between companies [27,40,41]. The consequence of these changes, and the risks arising from them, is also a growing social awareness. The problem is the fear of job loss due to the introduction of new technologies [27] and their influence on the environment [42].

**Fig 1:**
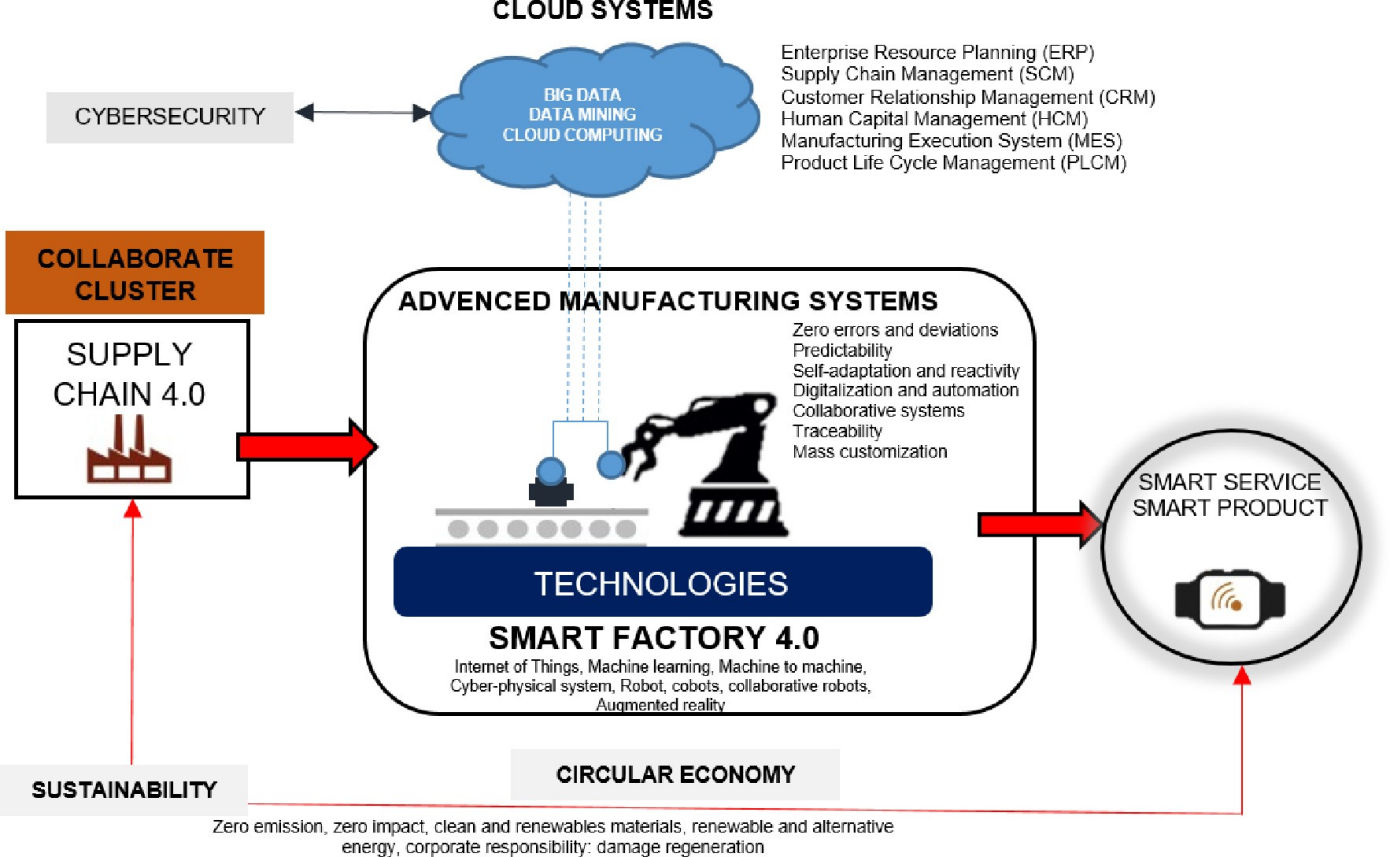
The self-similar industrial ecosystems in Industry 4.0. (Own elaboration based on [44]).

The integration of industrial systems is very important in this regard. The use of the Internet of things, artificial intelligence, cloud computing and the maximization of human-machine cooperation (robots, cobots and collaborative robots) creates intelligent and self-organizing systems that can efficiently and effectively carry out the production tasks assigned to them [43] (Fig 1).

These processes also enable the integration of distributed (logically or geographically) production systems, including services. This refers to the creation of smart factories that, as autonomous, self-regulating, integrated and flexible units, will have the capacity to adapt very quickly to production needs as well as market, environmental, social, and climate requirements [45].

Therefore, it can be claimed that in order to manage these changes, individual countries or groups of countries need to take actions as soon as possible to adapt to the digital transformation that is already underway.

### 2.3. Readiness for Industry 4.0 in the EU countries

In the case of the EU, European institutions, and the possibility of cooperation between individual countries, are of great importance in both promoting and implementing the digital economy. In order for this cooperation to be effective and bring the expected results, it is necessary to target it in accordance with the specificity and diversity of individual EU countries. It is also important to know the current state of knowledge and the level of development of the digital economy in these countries.

In terms of publications on digitalization processes and the idea of Industry 4.0, many studies can be found [45–60], and their number is growing rapidly. The presented literature review focused only on the most relevant works on this subject. This review aimed to get acquainted with the studies that looked at the processes of digitalization, automation and robotization of enterprises, sectors and countries, as well as methods used to evaluate these processes so far.

Nick et al. [46] in their study presented the research results related to the readiness of Hungarian companies to implement the concept of Industry 4.0. For this purpose, they conducted a study using a survey questionnaire consisting of 99 questions on issues related to the collection, processing and use of data on production processes, machines and products. It was found that most companies collect data about their production but do not use them in the later stages of these processes. On the other hand, in another paper, Gürdür et al. [47] assessed the readiness of Swedish industry to analyze data. The paper presents the results of a survey on the adoption and use of data analytics in the Swedish industry, with the aim of digitalization. The survey was conducted with a sample of more than 100 respondents from manufacturing, technology, engineering, telecommunications and automotive industries in Sweden. The results showed that Swedish industry has a high rate of resource readiness for data analysis as well as the necessary tools and human resources. Also, the cultural readiness level of Sweden, which includes the approval of data-driven decision making, was rated between high and very high. In terms of the readiness level of information systems, it was reported to be between medium and high, with the exception of telecommunications. The level of organizational readiness was found to be between medium and low. All in all, long organizational delays were shown to limit the broad applicability of data analysis in business.

Majstorović et al. [48] assessed the readiness of production companies in Serbia to implement the idea of Industry 4.0. In this article, they presented a model for assessing the maturity and readiness of production organizations (industries) to operate and implement Industry 4.0 in their environment. Castro et al. [49] developed a tool to assess the level of readiness of Portuguese companies for the digitalization process of the economy. For this purpose, they developed a tool that assesses the readiness level of the i4.0.—SHIFTo4.0 company. It is based on a model that considers six different dimensions: strategy and organization; smart factory; smart operations; smart products; data-driven services; and human resources. Each dimension is ranked on a scale between 0 and 5. The results of the analysis include a report with a set of recommendations, or a roadmap, to help the company implement i4.0 to achieve a higher level of readiness. Kopp and Basl [50] studied the readiness of Czech companies to implement the idea of Industry 4.0. The study examined as to whether Czech companies were interested in the fourth industrial revolution and as to whether they were ready for change. The results showed that some companies were already committed to these changes. However, their number was found to be insufficient. This is probably due to the fact that Industry 4.0 requires considerable investment from the very beginning. Tortorella et al. [39] examined the relationship between lean production and the implementation of Industry 4.0 in Brazilian production companies. For this purpose, they used data from a study of 110 companies of different sizes and industries, at different stages of LP implementation. The collected data were analyzed using a multivariate analysis. The results indicate that LP is positively related to the Industry 4.0 technologies, and their simultaneous implementation leads to greater productivity improvements.

Muscio and Ciffolilli [51] investigated factors influencing the ability to integrate different technologies of Industry 4.0. In particular, they examined the role of European funding and networking in relation to the ability to develop Industry 4.0 by integrating different enabling technologies. The findings indicate that EU funds, such as FP7, can facilitate technological development by sharing knowledge among multiple regional actors/entities and by promoting the gathering of physical and human resources. These funds and network centrality are significantly related to the ability to integrate the enabling technologies of Industry 4.0.

Nhamo et al. [52] utilized the min-max method to investigate the readiness of 212 countries and regions to implement the idea of Industry 4.0. They used ICT-related sustainability indicators. The results showed that the top 10 countries scored between 71.27 and 78.26 points out of 100, while the 10 countries with the lowest scores (all African countries) scored between 0.02 and 5.80 points. In terms of regions, the European Union ranked first with a score of 60.20 points and Sub-Saharan Africa was last with 13.04 points. Castelo-Branco et al. [53] investigated factors characterizing Industry 4.0 in relation to production processes in the EU-22 countries. The analysis proved that the existence of digital infrastructure, combined with analytical capabilities for handling big data sets, makes it possible to achieve a high level of readiness for Industry 4.0. At the EU level, five homogeneous groups of countries were defined, with different analytical capabilities for handling big data sets.

When analyzing the presented publications, it can be stated that they mainly concern the readiness of companies, sectors or countries to implement the idea of Industry 4.0. These works present interesting research results and many valuable remarks and hints concerning the whole process of digitalization.

The process of digitalization of enterprises is also associated with the use of robots in production processes, among other activities. In one study [67], the author attempted to provide answers to the following question: What drives and hinders robotization in Central and Eastern Europe? The research results showed, among other findings, that in these countries, the implementation of robots is highly concentrated in one sector, which is the automotive industry. By contrast, in other European countries there is more sectoral diversity in the robotization process. Turja and Oksanen [68], on the other hand, conducted a study on the acceptance of robots by employees in the EU-27 countries. The results indicate that in terms of robot acceptance, these countries can be divided into conventional and innovative ones. Positive attitudes toward robots were found to be higher in countries with a high rate of implementation of new technologies and work automation.

Several papers also address the issue of determining indicators to assess the level of digital transformation. From the point of view of the present work, these are very valuable publications. Based on these publications [27,32,54–57], a set of determinants (indicators) was selected to assess the level of digitalization and robotization of enterprises in the context of Industry 4.0 and similarity analysis of the EU countries. These indicators are presented and discussed in Section 2.2.

In the publications to date, there is a noticeable lack of a comprehensive approach to assessing the level of digitalization and robotization of individual EU countries with a broad consideration of the areas that determine this level. Also, no reference to the size of enterprises could be found, which is of great importance for the implementation of these processes. Practice shows clear differences in the implementation of innovative solutions depending on the size of an enterprise.

Therefore, it can be concluded that the conducted literature search fully justifies taking up this subject matter and posing the following research questions:

1. What is the level of digitalization and robotization across the EU countries?
2. What is the level of digitalization and robotization among enterprises in the EU countries according to their size?
3. What is the similarity between the EU countries in terms of the digitalization and robotization of enterprises of these countries, taking into account the size of these enterprises?

The use of modern tools for this analysis in the form of the TOPSIS method and the Kohonen’s artificial networks makes the findings credible and allowed the authors to gain new knowledge in this area. However, the developed and applied research methodology for assessing the level of development of the EU countries in the field of digitalization and robotization of enterprises and the division of these countries into similar groups is a new approach to this issue.

## 3. Materials and methods

The study, which aimed to assess the level of digitization and robotization of enterprises (in total, and divided into small, medium and large enterprises) in the EU-27 countries and to identify the similarity of these countries, was carried out using data from the Eurostat database, the TOPIS method and the Kohonen’s neural networks.

### 3.1. Data

In order to assess the level of digitalization and robotization of enterprises in the EU countries and make a comparative similarity analysis for the EU countries, data from the Eurostat database for the year 2018 were used [69].

Data used in the study concern the ICT usage and e-business in enterprises in the EU countries. Based on these data, specific indicators (determinants) were defined and used to conduct the presented analysis. These data concern enterprises classified according to the NACE Rev. 2 [70].

In order to assess the level of digitalization and robotization in the EU countries and to determine the similarity between these countries, it was necessary to choose appropriate determinants. On the basis of the literature search, 8 areas were selected, which have a decisive influence on the course of digitalization and robotization processes in the EU countries. These areas are: big data analysis, cloud computing, 3D printing, robotics, integration of internal processes, integration with customers/suppliers, supply chain management, Internet accessibility and digital skills (ICT training).

Descriptions of the determinants adopted for evaluation that characterize the processes of the digitalization and robotization of enterprises in the EU countries are presented in Table 1.

**Table 1:** Determinants characterizing the processes of digitalization and robotization of enterprises in the EU countries.

Area	Determinants	Marking	Description
Big data analysis	Analysis of big data from any data source, % of enterprises	X1	Big data analysis technologies currently play a huge role in the introduction of all kinds of innovative solutions. The development of digital technologies is based on the analysis of large data sets.
	Analyse own big data from enterprise’s smart devices or sensors, % of enterprises	X2	
	Analysis of big data from geolocation of portable devices, % of enterprises	X3	
Cloud computing	Purchase of cloud computing services used over the internet, % of enterprises	X4	The use of cloud computing brings many benefits to enterprises. First of all, it lowers operating costs (reduces the cost of maintaining IT infrastructure) and increases data security. Cloud technologies enable, among others, the storage of data, applications, programs, as well as their operation from any place in the world (only Internet access is required).
	Purchase of computing power to run the enterprise’s own software, % of enterprises	X5	
	Buy high CC services, % of enterprises	X6	
3D printing	Use of 3D printing, % of enterprises	X7	3D printing technology is currently one of the most rapidly growing fields related to the digitalization of the global economy. 3D printing technology allows companies to complete the full cycle of product manufacturing in a short time, which in many cases is their competitive advantage.
Robotics	Use of industrial robots, % of enterprises	X8	Robotization of production processes involving the replacement of human activities with machines is the quintessential process of digitalization of the economy. The introduction of robotization is associated with quite high costs, but the advantages of this process are enormous.
Integration of internal processes	Enterprises that have ERP software, % of enterprises	X9	ERP applications (systems) in companies are designed to facilitate the flow of information and the possibility of horizontal and vertical integration. This is mainly due to the integration of processes related to business planning, purchase of goods and services, marketing processes, sales, enterprise-consumer relations, company finances, and human resources.
	Enterprises using software solutions like Customer Relationship Management, % of enterprises	X10	
Integration with customers/suppliers, supply chain management	Enterprises sending eInvoices, suitable for automated processing, % of enterprises	X11	Supply chain management includes all activities related to the exchange of information between an enterprise and its suppliers and/or customers. The digitalization of this area of the companies’ activities is crucial for the optimization of their operations.
	Enterprises receiving eInvoices, suitable for automated processing, % of enterprises	X12	
	Enterprises whose business processes are automatically linked to those of their suppliers and/or customers, % of enterprises	X13	
Internet connectivity	The maximum contracted download speed of the fastest fixed internet connection is at least 100 Mb/s, % of enterprises	X14	Businesses’ access to and use of the Internet is absolutely fundamental to their operations in the digital economy. The possibility of faster transfer of information and more effective ways of using it allow to leapfrog improvements in the effectiveness and efficiency of the enterprise, practically in all areas of its operations.
	Enterprises connecting to the internet via a mobile broadband connection (3G modem or 3G handset), % of enterprises	X15	
Digital skills (ICT training)	Enterprises that provided training to develop/upgrade ICT skills of their personnel, % of enterprises	X16	The introduction of digital technologies is associated with the need to build a digital society. Currently, digital skills are considered to be a basic condition that determines the possibility of developing a digital economy. They require continuous upgrading of skills by employees, so that with the development and implementation of new technologies there is no phenomenon of digital exclusion.

### 3.2. The TOPSIS method

In order to assess the level of digitalization and robotization of enterprises in the EU countries, the Technique for Order Preference by Similarity to an Ideal Solution (TOPSIS) method was applied. This method belongs to the group of multi-criteria decision-making methods [71,72]. According to this method, the selection of the best solution is based on the evaluation of a set of criteria. The basic assumption is to identify the best solution among a finite set of solutions (objects). The TOPSIS method is widely used in many areas of economy [73]. It is based on a comparison of decision variants with some points, the so-called reference solutions (ideal solution and anti-ideal solution). These comparisons aim at ordering these variants (the closer a variant is to the ideal solution and further from the anti-ideal solution, the higher it is located).

The general algorithm of the research procedure using the TOPSIS method was to:

1. construct the decision matrix:
$$X=(x_{ij})$$ (1)
  where: *x*_*ij*_∈ℝ;
2. calculate the normalized decision matrix:
$$x_{ij}=\frac{x_{i}}{\sqrt{\sum_{i=1}^{m}{x^{2}}_{i}}}$$ (2)
3. calculate the normalized decision matrix taking into account the weights for each criterion, according to the following relationship:
$$v_{ij}=x_{ij}∙w_{j}$$ (3)
  where: *w*_*j*_ is the weight of the *j*-th criterion; $\sum_{j=1}^{n}w_{j}=1$
4. calculate the vector of values of the ideal solution S+ and the anti-ideal solution S ^*-*^:
$$S^{+}=(x_{1}^{+},x_{2}^{+},x_{3}^{+},\ldots x_{n}^{+})=\{({max}_{i}x_{ij}|j\in B),({min}_{i},x_{ij}|j\in C)\}$$ (4)
$$S^{-}=(x_{1}^{-},x_{2}^{-},x_{3}^{-},\ldots x_{n}^{-})=\{({min}_{i}x_{ij}|j\in B),({max}_{i},x_{ij}|j\in C)\}$$ (5)
  The ideal solution contains the highest maximized and lowest minimized variables. The anti-ideal solution is constructed inversely.
5. calculate the value of the distance ($d_{i}^{+}\;and\;d_{i}^{-}$) of each considered solution from the ideal solution *S*^*+*^ ($d_{i}^{+}$) and the anti-ideal solution *S*^*-*^ ($d_{i}^{-}$) (6 and 7):
$$d_{i}^{+}=\sqrt{\sum_{j=1}^{n}{\left(x_{ij}-x_{j}^{+}\right)}^{2}}$$ (6)
$$d_{i}^{-}=\sqrt{\sum_{j=1}^{n}{\left(x_{ij}-x_{j}^{-}\right)}^{2}}$$ (7)
  where $d_{i}^{+}$ is the distance from the ideal solution and $d_{i}^{-}$ is the distance from the anti-ideal solution.
6. identify the ranking coefficient that determines the similarity of objects to the ideal solution (8):
$$P_{i}=\frac{d_{i}^{-}}{d_{i}^{+}+d_{i}^{-}}\;\mathrm{for}\;i=1,2,\ldots ,m,\;\mathrm{wherein}\;0\leq P_{i}\leq 1$$ (8)

In the last stage of the procedure, the solutions are ranked with respect to the value of the coefficient of relative closeness of the objects to the ideal object—*Pi*. The highest *Pi* value indicates the best solution for the considered linear ordering problem.

Based on the analysis, the EU countries were divided into 4 classes. The first class included countries with the highest level of digitalization and robotization, and the fourth class included countries with the lowest.

The criteria for dividing the EU countries in terms of the level of digitalization and robotization of production enterprises into different classes were as follows:

1. Class 1: High level of digitalization and robotization:
$$P_{i}\geq \bar{P_{i}}+s_{P_{i}}$$ (9)
2. Class 2: Medium-high level of digitalization and robotization:
$$\bar{P_{i}}+s_{P_{i}}>P_{i}\geq \bar{P_{i}}$$ (10)
3. Class 3: Medium-low level of digitalization and robotization:
$$\bar{P_{i}}>P_{i}\geq \bar{P_{i}}-s_{P_{i}}$$ (11)
4. Class 4: Low level of digitalization and robotization:
$$\bar{P_{i}}>P_{i}\geq \bar{P_{i}}-s_{P_{i}}$$ (12)
  where *P*_*i*_ is the TOPSIS measure, $\bar{P_{i}}$ is the mean value of TOPSIS measure and $s_{P_{i}}$ is the standard deviation.

### 3.3. The Kohonen’s artificial neural networks

**Fig 2:**
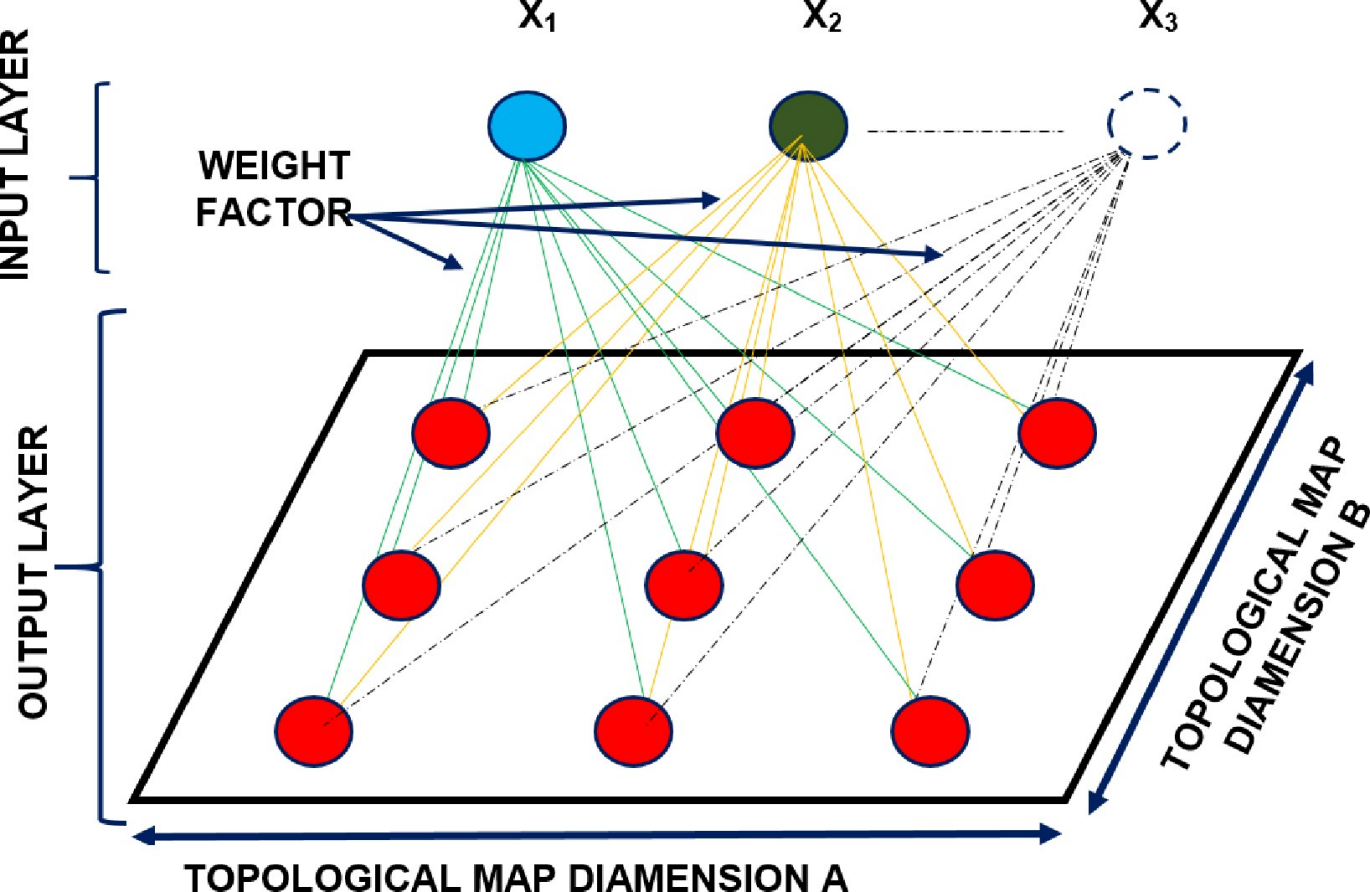
Kohonen’s neural network scheme.

The Kohonen’s artificial neural networks belong to the self-organizing group, in which learning is carried out by a self-organizing method of competitive type [74]. It consists in feeding signals to the inputs of the network and then selecting through competition the winning neuron that best matches the input vector [75,76]. The main task of the Kohonen’s network is to organize multidimensional information, so that it can be presented and analyzed in a space with much fewer dimensions—e.g., a two-dimensional topological map. The general model of the Kohonen’s network is shown in Fig 2.

The network learning algorithm proposed by Kohonen consists of the following procedural steps:

1. for each learning vector, the neuron closest to the input learning vector is localized. This neuron is called the winner (***w***):
$$w=arg\left({min}_{x\in \{x_{1},\ldots ..x_{i}\}}(d(v,x))\right)$$ (13)
  where:
$$d(v_{j},x_{i})=\sqrt{\sum_{i-1}^{n}{\left(v_{j}-x_{i,}\right)}^{2}}$$ (14)
2. the winner neuron (*w*) is assigned with all *n*_i_, neurons that have a neighborhood relationship with it. The set of these neurons is called the neighborhood.
3. The winner’s weight vector is updated according to equation:
w=w+β∙(v−w) (15)
  where *β ∈* [0,1] is learning rate.
4. the weight vectors neighboring with the winner neuron are updated according to equation:
$$n_{i}=n_{i}+g(neighborhood)∙\beta ∙(v-n_{i})$$ (16)
  where: *g* (neighborhood) is a function that calculates the learning rate modification for a neighborhood (closer neighborhood should learn more than more distant neighborhood).

In order to carry out the identification of groups of similar countries in terms of digitalization and robotization, it was necessary to normalize them according to the normalization equation for stimulant:

$$x_{s}=\frac{x_{ij}-\min\;x_{i}}{\max\;x_{i}-\min\;x_{i}}$$ (17)

The normalization process made it possible to present the profiles of the EU countries, in terms of digitalization and robotization, located in each cluster in a graphical form.

## 4. Results

The conducted research was divided into preliminary and fundamental. The result of the preliminary part was the determination of statistical parameters of indicators adopted for the study. The result of the fundamental research was the division of the EU countries into groups based on the level of digitalization and robotization of enterprises in these countries (using the TOPSIS method) and their division into similar groups (using the Kohonen’s method).

### 4.1. The preliminary statistical analysis of indicators on digitalization and robotization of the EU enterprises

**Fig 3:**
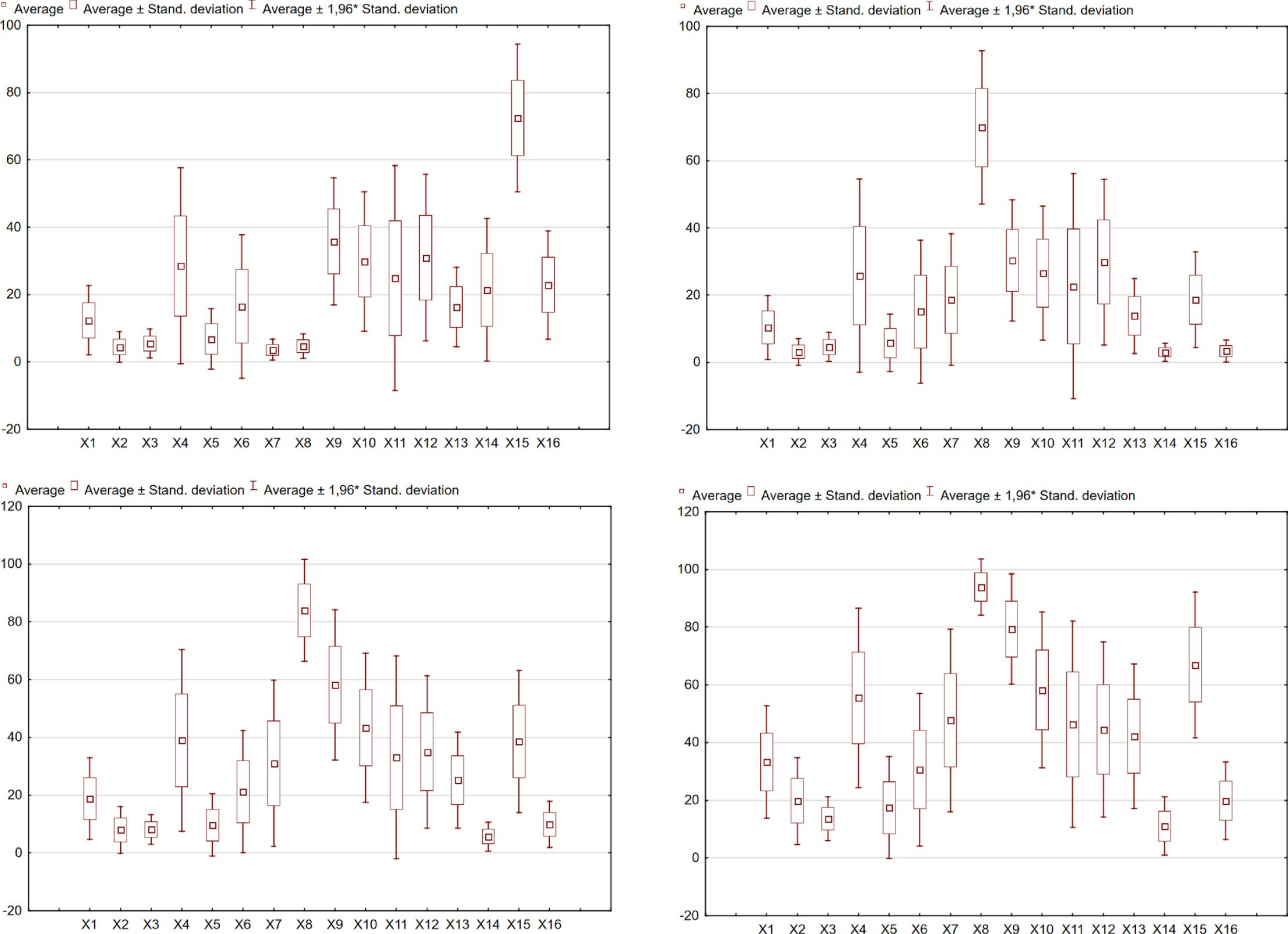
Basic descriptive statistics for the determinants of digitization and robotization of enterprises in the EU27 countries (a-all enterprises, b-small enterprises, c-medium enterprises, d-large enterprises).

The indicators used in the study, characterizing 8 main areas of the level of the EU countries in the field of digitalization and robotization, were preliminarily processed and their basic statistical parameters were determined, which are summarized in Tables 2–5 and Fig 3. These indicators were determined for small, medium and large-sized enterprises and in general.

**Table 2:** Statistical parameters of indicators determining the level of the EU countries in terms of digitalization and robotization of all enterprises.

Variable	Mean	Median	Min	Max	Standard deviation	Coefficient of variation, %	Skewness	Kurtosis	Percentile 10	Percentile 90
X1	12.33	11.00	5.00	24.00	5.23	42.43	0.66	-0.43	6.00	20.00
X2	4.41	4.00	2.00	10.00	2.29	51.99	0.84	-0.17	2.00	8.00
X3	5.44	5.00	2.00	9.00	2.19	40.22	0.24	-1.26	3.00	9.00
X4	28.52	25.00	8.00	65.00	14.88	52.18	0.96	0.33	11.00	56.00
X5	6.81	6.00	1.00	21.00	4.57	67.13	1.41	2.29	2.00	13.00
X6	16.48	13.00	4.00	44.00	10.91	66.17	1.22	0.57	5.00	37.00
X7	21.37	19.00	4.00	46.00	10.78	50.45	0.72	0.08	9.00	37.00
X8	72.48	73.00	47.00	94.00	11.21	15.47	-0.50	0.52	51.00	83.00
X9	35.78	35.00	14.00	53.00	9.59	26.81	-0.13	-0.45	23.00	48.00
X10	29.89	31.00	12.00	56.00	10.56	35.31	0.44	-0.10	17.00	44.00
X11	24.89	20.00	7.00	79.00	17.09	68.66	1.83	3.29	10.00	55.00
X12	30.96	30.00	12.00	70.00	12.62	40.75	0.97	2.04	17.00	46.00
X13	16.26	16.00	6.00	30.00	6.02	37.04	0.53	0.03	9.00	26.00
X14	3.63	4.00	1.00	7.00	1.60	43.99	0.23	-0.60	2.00	6.00
X15	22.85	23.00	6.00	37.00	8.21	35.94	-0.17	-0.77	11.00	32.00
X16	4.67	5.00	1.00	8.00	1.90	40.74	-0.10	-0.80	2.00	7.00

**Table 3:** Statistical parameters of indicators determining the level of the EU countries in terms of digitalization and robotization of small enterprises.

Variable	Mean	Median	Min	Max	Standard deviation	Coefficient of variation, %	Skewness	Kurtosis	Percentile 10	Percentile 90
X1	10.44	9.00	3.00	21.00	4.87	46.63	0.51	-0.70	5.00	18.00
X2	3.19	3.00	1.00	8.00	2.02	63.41	0.94	0.10	1.00	7.00
X3	4.59	4.00	1.00	9.00	2.22	48.41	0.29	-0.98	2.00	8.00
X4	25.81	21.00	7.00	62.00	14.64	56.73	1.04	0.42	9.00	54.00
X5	5.78	5.00	1.00	20.00	4.37	75.65	1.57	3.08	1.00	12.00
X6	15.11	11.00	3.00	42.00	10.87	71.94	1.23	0.53	4.00	36.00
X7	18.67	17.00	3.00	41.00	9.98	53.45	0.76	0.01	8.00	36.00
X8	69.93	70.00	44.00	93.00	11.65	16.66	-0.39	0.48	48.00	81.00
X9	30.33	29.00	10.00	47.00	9.21	30.37	-0.05	-0.60	20.00	43.00
X10	26.56	26.00	10.00	52.00	10.15	38.24	0.52	-0.04	15.00	40.00
X11	22.63	17.00	6.00	78.00	17.09	75.52	1.92	3.66	8.00	53.00
X12	29.85	29.00	11.00	68.00	12.54	42.01	0.92	1.77	16.00	43.00
X13	13.81	13.00	5.00	27.00	5.72	41.44	0.67	0.18	7.00	23.00
X14	3.00	3.00	1.00	6.00	1.36	45.29	0.30	-0.48	1.00	5.00
X15	18.59	18.00	5.00	31.00	7.27	39.11	-0.02	-0.92	8.00	27.00
X16	3.37	3.00	1.00	7.00	1.64	48.79	0.53	-0.09	1.00	5.00

**Table 4:** Statistical parameters of indicators determining the level of the EU countries in terms of digitalization and robotization of medium-sized enterprises.

Variable	Mean	Median	Min	Max	Standard deviation	Coefficient of variation, %	Skewness	Kurtosis	Percentile 10	Percentile 90
X1	18.85	17.00	8.00	37.00	7.20	38.19	1.05	0.56	12.00	32.00
X2	7.96	7.00	3.00	19.00	4.11	51.60	0.96	0.53	3.00	14.00
X3	8.15	8.00	4.00	13.00	2.61	32.06	0.24	-0.79	5.00	12.00
X4	39.04	36.00	13.00	77.00	16.06	41.13	0.68	0.17	19.00	62.00
X5	9.70	8.00	2.00	23.00	5.48	56.43	0.89	0.48	3.00	17.00
X6	21.22	18.00	6.00	50.00	10.82	50.99	1.09	0.88	7.00	37.00
X7	31.11	28.00	12.00	69.00	14.67	47.14	1.07	1.06	14.00	55.00
X8	84.00	86.00	61.00	99.00	9.03	10.75	-0.68	0.49	70.00	94.00
X9	58.22	61.00	30.00	75.00	13.24	22.74	-0.54	-0.66	40.00	74.00
X10	43.33	45.00	22.00	71.00	13.19	30.45	0.03	-0.76	23.00	60.00
X11	33.07	27.00	11.00	85.00	17.91	54.16	1.45	1.82	17.00	63.00
X12	35.04	36.00	16.00	80.00	13.46	38.43	1.36	3.65	20.00	47.00
X13	25.26	24.00	9.00	42.00	8.45	33.44	0.01	-0.51	13.00	36.00
X14	5.67	5.00	2.00	11.00	2.53	44.59	0.45	-0.76	3.00	9.00
X15	38.63	39.00	10.00	59.00	12.55	32.48	-0.44	-0.03	17.00	57.00
X16	9.85	10.00	1.00	17.00	4.07	41.35	-0.22	-0.62	4.00	15.00

**Table 5:** Statistical parameters of indicators determining the level of the EU countries in terms of digitalization and robotization of large enterprises.

Variable	Mean	Median	Min	Max	Standard deviation	Coefficient of variation, %	Skewness	Kurtosis	Percentile 10	Percentile 90
X1	33.30	31.00	17.00	55.00	9.95	29.88	0.67	-0.24	23.00	48.00
X2	19.81	17.00	9.00	35.00	7.68	38.74	0.81	-0.45	11.00	34.00
X3	13.59	14.00	6.00	21.00	3.89	28.59	-0.06	-0.59	8.00	19.00
X4	55.52	55.00	25.00	92.00	15.88	28.59	0.43	0.09	39.00	79.00
X5	17.44	14.00	6.00	38.00	9.01	51.63	0.89	-0.08	7.00	34.00
X6	30.67	28.00	11.00	58.00	13.50	44.04	0.69	-0.31	15.00	55.00
X7	47.70	47.00	23.00	86.00	16.16	33.87	0.79	0.46	29.00	77.00
X8	93.89	95.00	79.00	100.00	4.98	5.30	-1.42	2.17	85.00	99.00
X9	79.33	82.00	59.00	92.00	9.73	12.27	-0.66	-0.60	62.00	90.00
X10	58.22	56.00	34.00	80.00	13.79	23.69	0.05	-0.95	42.00	78.00
X11	46.33	42.00	19.00	90.00	18.22	39.33	0.70	-0.24	27.00	74.00
X12	44.48	44.00	18.00	90.00	15.50	34.84	0.87	1.67	28.00	65.00
X13	42.26	41.00	20.00	66.00	12.79	30.27	0.28	-0.76	25.00	61.00
X14	11.07	11.00	3.00	21.00	5.19	46.85	0.09	-1.23	4.00	17.00
X15	66.96	67.00	30.00	88.00	12.88	19.23	-0.92	1.73	53.00	81.00
X16	19.81	21.00	3.00	32.00	6.85	34.57	-0.61	0.46	8.00	28.00

Based on the results, it can be concluded that the presented sets of indicators are characterized by a wide range of the coefficient of variation. Thus, the condition of diagnostic features is met, which should be marked with a significant differentiation within the studied community.

The determination of the skewness coefficient was aimed at assessing the asymmetry of the distribution of variables. The values of this coefficient for determinants X1 (Analysis of big data from any data source), X3 (Analysis of big data from geolocation of portable devices), X4 (Purchase of cloud computing services used over the internet), X5 (Purchase of computing power to run the enterprise’s own software), X6 (Purchase of high CC services (accounting software applications, CRM software, computing power), X7 (Use of 3D printing), X8 (Use of industrial robots), X9 (Enterprises that have ERP software), X10 (Enterprises using software solutions like Customer Relationship Management), X11 (Enterprises sending eInvoices, suitable for automated processing), X12 (Enterprises receiving eInvoices, suitable for automated processing), X13 (Enterprises whose business processes are automatically linked to those of their suppliers and/or customers), X14 (The maximum contracted download speed of the fastest fixed Internet connection is at least 100 Mb/s) were found to be positive for all study groups (companies), which indicates the right-sided asymmetry of the distribution. This, in turn, indicates that in most EU countries the values of these indicators were lower than the mean value for the EU-27. The positive value of the skewness coefficient was also present for determinant X2 (Analysis of own big data from enterprise’s smart devices or sensors).

All used determinants meet the condition of diagnostic variables, which means that they are characterized by significant variation, determined by the value of the coefficient of variation.

The use of kurtosis makes it possible to assess the concentration of results around the mean value. The distribution of indicators for which the value of the kurtosis coefficient is positive is concentrated near the mean value. By contrast, when these values are negative, there is a greater dispersion of these indicators.

### 4.2. Determining the level of digitization and robotization of EU countries

In the first stage of the fundamental research, the use of the TOPSIS linear ordering method with a synthetic measure of the degree of similarity to the pattern allowed for the determination of the ranking of studied EU countries in terms of the level of digitalization and robotization of their enterprises. All diagnostic variables adopted for the study were stimulants. In the analysis, all these indicators were assigned the same value of weights. The results of calculating the measure of distance of the EU-27 countries from the pattern and anti-pattern, the values of the TOPSIS *Pi* synthetic measure for the analyzed years and the classification of the EU-27 countries in terms of the level of digitalization and robotization of their enterprises are presented in Table 6.

**Table 6:** Distance of the EU-27 countries from the pattern and anti-pattern together with the TOPSIS measure for the level of digitalization of individual groups of enterprise.

	All enterprises, without financial sector (10 persons employed or more)				Small enterprises (10–49 persons employed), without financial sector				Medium enterprises (50–249 persons employed), without financial sector				Large enterprises (250 persons employed or more), without financial sector			
	*S*_i_^+^	*S*_i_^-^	*P*_*i*_	Ranking	*S*_i_^+^	*S*_i_^-^	*P*_*i*_	Ranking	*S*_i_^+^	*S*_i_^-^	*P*_*i*_	Ranking	*S*_i_^+^	*S*_i_^-^	*P*_*i*_	Ranking
Belgium	0.080	0.547	0.873	3	0.395	0.722	0.647	4	0.227	0.560	0.712	5	0.080	0.547	0.873	3
Bulgaria	0.521	0.085	0.141	23	1.418	0.086	0.057	25	0.947	0.077	0.075	25	0.521	0.085	0.141	23
Czech Republic	0.378	0.176	0.317	16	1.151	0.107	0.085	21	0.708	0.171	0.195	18	0.378	0.176	0.317	16
Denmark	0.038	0.548	0.935	2	0.235	1.014	0.812	2	0.183	0.724	0.798	2	0.038	0.548	0.935	2
Germany	0.279	0.222	0.443	10	0.971	0.308	0.241	14	0.583	0.271	0.317	13	0.279	0.222	0.443	10
Estonia	0.291	0.166	0.363	13	1.044	0.161	0.134	17	0.576	0.165	0.223	17	0.291	0.166	0.363	13
Ireland	0.211	0.263	0.555	7	0.601	0.474	0.441	6	0.353	0.365	0.509	6	0.211	0.263	0.555	7
Greece	0.551	0.047	0.078	26	1.429	0.085	0.056	26	0.796	0.096	0.108	23	0.551	0.047	0.078	26
Spain	0.293	0.166	0.362	14	0.833	0.320	0.277	12	0.488	0.249	0.338	11	0.293	0.166	0.362	14
France	0.238	0.217	0.477	8	0.905	0.309	0.255	13	0.504	0.259	0.339	10	0.238	0.217	0.477	8
Croatia	0.491	0.077	0.136	24	0.972	0.190	0.164	16	0.722	0.132	0.154	20	0.491	0.077	0.136	24
Italy	0.334	0.146	0.305	17	1.020	0.204	0.167	15	0.640	0.225	0.260	16	0.334	0.146	0.305	17
Cyprus	0.616	0.072	0.105	25	1.310	0.119	0.083	22	0.734	0.164	0.182	19	0.616	0.072	0.105	25
Latvia	0.495	0.089	0.152	22	1.375	0.111	0.075	24	0.939	0.080	0.078	24	0.495	0.089	0.152	22
Lithuania	0.329	0.139	0.296	18	0.661	0.448	0.404	7	0.484	0.236	0.328	12	0.329	0.139	0.296	18
Luxembourg	0.362	0.139	0.278	19	0.834	0.325	0.281	11	0.566	0.203	0.264	15	0.362	0.139	0.278	19
Hungary	0.448	0.102	0.185	21	1.268	0.074	0.055	27	0.896	0.062	0.065	26	0.448	0.102	0.185	21
Malta	0.336	0.218	0.394	12	0.665	0.439	0.398	8	0.482	0.314	0.394	8	0.336	0.218	0.394	12
Netherlands	0.149	0.360	0.707	5	0.377	0.758	0.668	3	0.215	0.601	0.736	3	0.149	0.360	0.707	5
Austria	0.280	0.228	0.449	9	1.160	0.144	0.111	18	0.617	0.221	0.264	15	0.280	0.228	0.449	9
Poland	0.348	0.162	0.318	15	1.264	0.118	0.085	21	0.807	0.115	0.125	21	0.348	0.162	0.318	15
Portugal	0.260	0.189	0.420	11	0.678	0.381	0.360	9	0.456	0.275	0.376	9	0.260	0.189	0.420	11
Romania	0.647	0.032	0.047	27	1.312	0.111	0.078	23	1.050	0.034	0.031	27	0.647	0.032	0.047	27
Slovenia	0.196	0.380	0.659	6	0.755	0.315	0.294	10	0.409	0.349	0.460	7	0.196	0.380	0.659	6
Slovakia	0.412	0.113	0.215	20	1.119	0.110	0.089	19	0.741	0.100	0.118	22	0.412	0.113	0.215	20
Finland	0.026	0.650	0.962	1	0.129	1.307	0.910	1	0.078	0.917	0.922	1	0.026	0.650	0.962	1
Sweden	0.093	0.466	0.833	4	0.529	0.641	0.548	5	0.244	0.647	0.726	4	0.093	0.466	0.833	4

**Fig 4:**
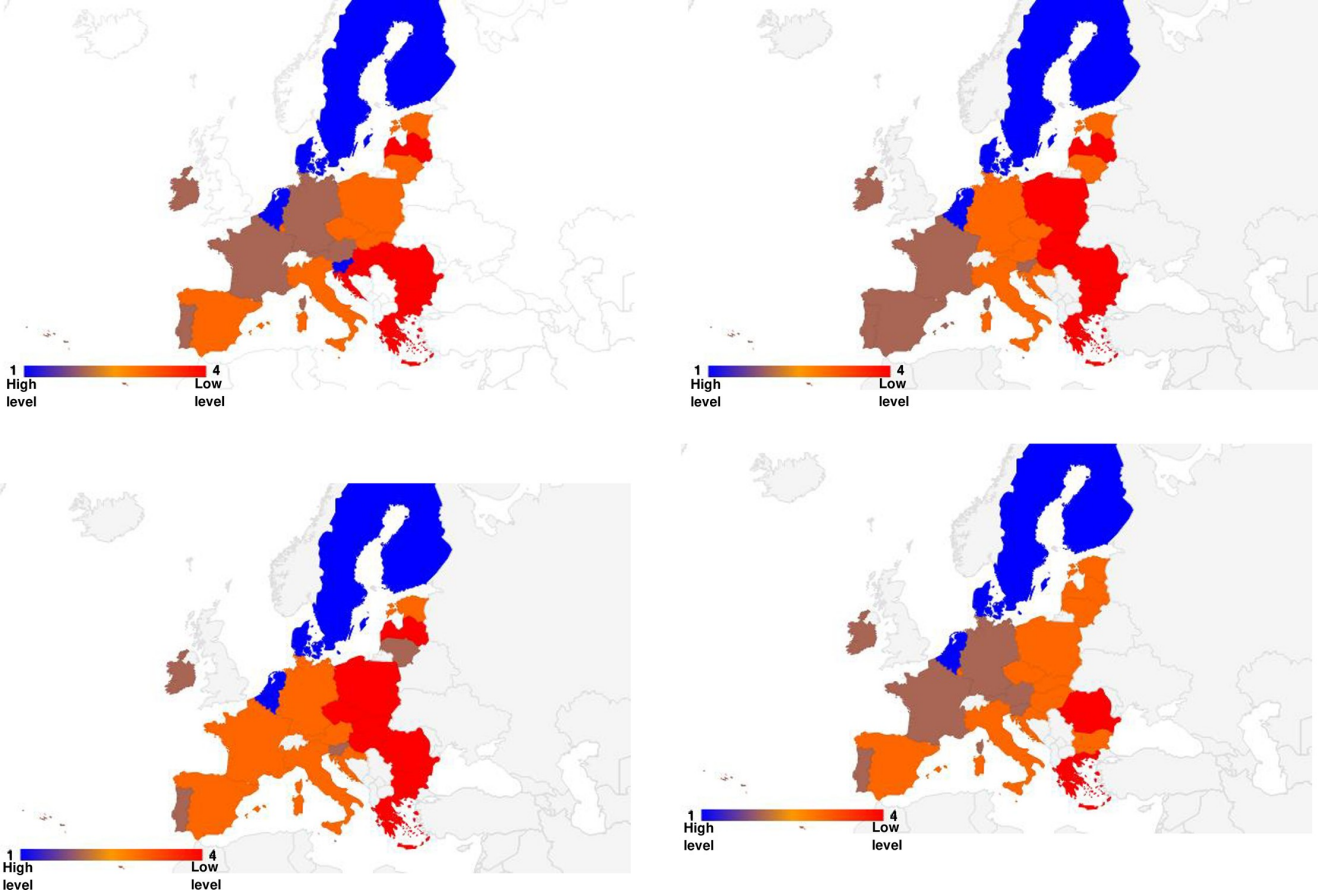
Division of the EU countries into similar classes in terms of the level of development of digitalization of enterprises (a- all enterprises, b–small enterprises, c-medium enterprises, d-large enterprises) (own elaboration).

In turn, Fig 4 presents the division of the EU-27 countries into classes (where: class 1 shows a high level, class 2 shows a medium-high level, class 3 shows a medium-low level and class 4 shows a low level) depending on the level of digitalization and robotization of their enterprises in total and taking into account their size.

The conducted analyses made it possible to classify the EU-27 countries into one of four classes in terms of the level of development of digitalization and robotization of their enterprises based on the adopted indicators.

Without taking into account the size of enterprises, the countries with a high level of development in this area are Finland, Denmark, Belgium, Sweden, the Netherlands and Slovenia. In the case of small, medium and large enterprises, the countries that have a high level of digitalization are Finland, Denmark, the Netherlands, Belgium, and Sweden.

The countries with medium-high levels of enterprise digitalization development without taking into account enterprise size are Ireland, France, Austria, Germany, and Portugal. By contrast, for small enterprises, these are Ireland, Lithuania, Malta, Portugal and Slovenia, and for medium-sized enterprises, these are Ireland, Slovenia, Malta, Portugal, France, and Spain. In terms of large enterprises, they are Slovenia, Ireland, France, Austria, Germany and Portugal.

The medium-low levels of digitalization and robotization of enterprises in total were found in the following countries: Malta, Estonia, Spain, Poland, Czechia, Italy, Lithuania, Luxembourg and Slovakia. For small enterprises, the countries include Luxembourg, Spain, France, Germany, Italy, Croatia, Estonia and Austria, and for medium-sized enterprises: Lithuania, Germany, Luxembourg, Austria, Italy, Estonia, Czechia, Cyprus and Croatia. Among large enterprises, medium-low levels of digitalization and robotization were found in the following countries: Malta, Estonia, Spain, Poland, Czechia, Italy, Lithuania, Luxembourg, Slovakia, Hungary, Latvia, Bulgaria, Croatia.

The low level of digitalization of enterprises without taking into account their size was found for Hungary, Latvia, Bulgaria, Croatia, Cyprus, Greece and Romania. For small enterprises, it was Slovakia, Czechia, Poland, Cyprus, Romania, Latvia, Bulgaria, Greece and Hungary and for medium-sized enterprises, it was Poland, Slovakia, Greece, Latvia, Bulgaria, Hungary and Romania. In terms of large enterprises, Cyprus, Greece and Romania are characterized by a low level of digitalization of enterprises.

In general, it can be said that in each analyzed group, the high level of digitalization was shown by enterprises from Finland, Denmark, Netherlands, Belgium and Sweden, the medium-high level–from Ireland and Portugal, the medium-low level–from Estonia, Luxembourg, Italy. The low level of digitalization, for each group, was found in Romania and Greece.

### 4.3. Dividing the EU countries into similar groups by the level of digitalization and robotization of enterprises using the Kohonen’s artificial neural networks

The second stage of fundamental research was to divide the EU countries into groups similar in terms of digitalization and robotization with the use of the Kohonen’s artificial networks. In batch learning, for proper model building, it is important to identify the correct number of columns and rows that define the size of a topological map. This size was determined with the following Eq (18) [77]:

$$k≅\sqrt{\frac{n}{2}}$$ (18)

where: *k* is the size of a topological map, *n* is the number of cases (countries).

**Fig 5:**
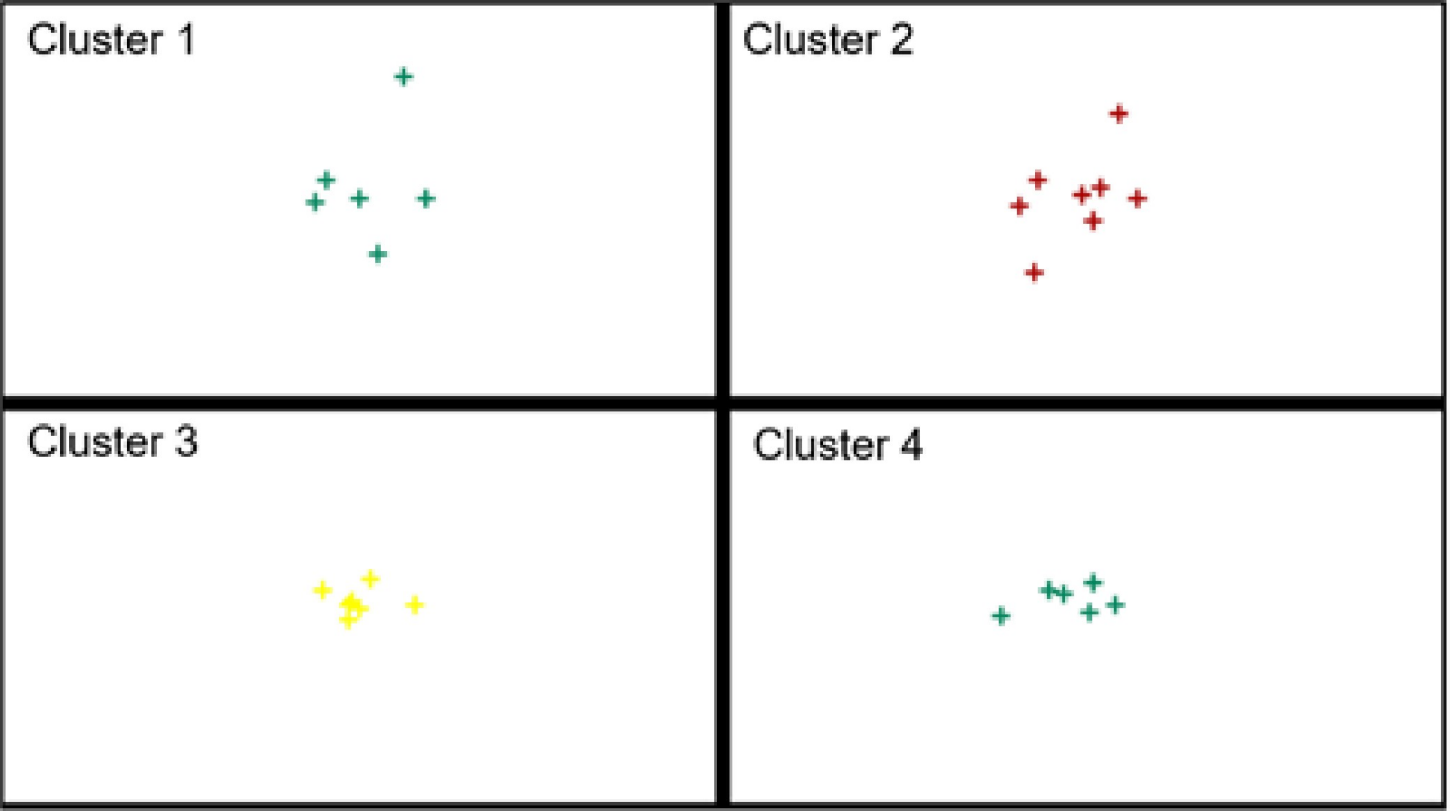
The topological map of similar groups of EU-27 countries for all enterprises.

**Fig 6:**
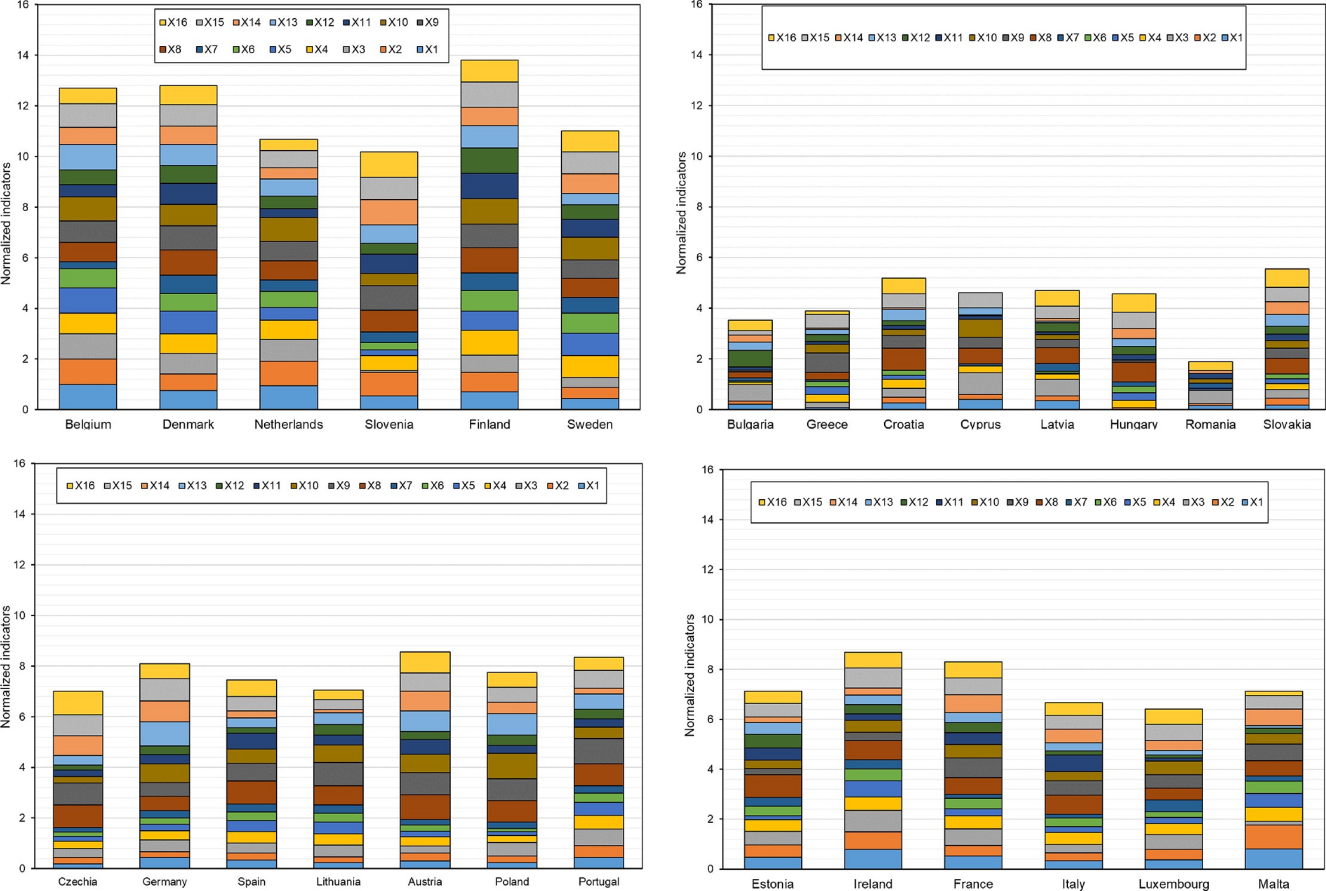
The summary of studied indicators on digitalization and robotization of enterprises for the EU countries in the designated similar groups (a-cluster 1, b-cluster 2, c-cluster 3, d-cluster 4).

The composition of clusters of similar countries in terms of the indicators adopted for the analysis for enterprises in total is presented in Table 7. The distribution of countries in the created clusters on the basis of the value of the activation function is presented on the topological map in Figs 5 and 6 shows the juxtaposition of the examined standardized indicators for the digitalization and robotization of enterprises in the EU countries in the determined similar groups.

**Table 7:** The division of the EU countries into similar groups based on the examined indicators for all enterprises together with activation values.

Elements of clusters 1	Activation function	Elements of clusters 2	Activation function	Elements of clusters 3	Activation function	Elements of clusters 4	Activation function
Belgium	0.80	Bulgaria	0.73	Czechia	0.73	Estonia	0.62
Denmark	0.47	Greece	0.74	Germany	0.67	Ireland	0.68
Netherlands	0.76	Croatia	0.59	Spain	0.50	France	0.52
Slovenia	1.10	Cyprus	0.91	Lithuania	0.62	Italy	0.59
Finland	0.66	Latvia	0.51	Austria	0.51	Luxembourg	0.59
Sweden	0.69	Hungary	0.81	Poland	0.53	Malta	0.89
		Romania	0.90	Portugal	0.58		
		Slovakia	0.57				

In order to highlight the similarity between countries within one cluster and differences with respect to other countries in other clusters, a uniform scale on the Y-axis was maintained. In the case of normalized values (Eq 17), the value of a single indicator ranges from 0 to 1. With a value of ’0’ the indicator does not appear in the profile, and with a value of ’1’ it indicates a state of perfect influence (Fig 6).

Based on the analysis, it can be concluded that cluster one includes six countries with a high level of digitalization and robotization of enterprises, namely Belgium, Denmark, Finland, Sweden, Netherlands and Slovenia (based on the TOPSIS analysis), and cluster two includes countries with a low level of this digitalization (Bulgaria, Greece, Croatia, Cyprus, Latvia, Hungary, Romania) and one country with a low medium level (Slovakia).

In the cluster analysis conducted using the Kohonen’s method, the division of countries into groups is based on the multidimensional similarity of the adopted indicators. This does not mean, however, that a given cluster consists of countries with only the highest, medium or lowest values of these indicators. The basis for the division is the similarity (homogeneity) of their structure in relation to all indicators (multidimensionality of classification). Therefore, different group divisions can be observed using the TOPSIS method and the Kohonen’s networks.

The EU27 countries (Table 7) located inside one cluster are most similar to each other in terms of indicators for assessing the process of digitalization and robotization of enterprises, and at the same time they are different from countries located in other clusters. By contrast, the EU countries inside the same cluster show the greatest similarity when they are as close to each other as possible.

When analyzing the distribution of the EU countries on the topological map (Fig 5) and the values of activation functions (Table 7), it can be concluded that the greatest internal differentiation was shown by the countries from cluster 1 and 2, and the most similar are countries from cluster 3 and 4. In cluster 1, Belgium and Denmark showed the greatest similarity, as well as the Netherlands, Slovenia and Sweden. Finland, which has the highest level of digitalization and robotization of enterprises among the EU countries, was found to differ the most from these countries. In cluster 4, the greatest differentiation from the other countries in this cluster was shown by Romania, which has been ranked in the lowest positions in Europe for years in terms of the digitalization and robotization of enterprises [78,79].

**Fig 7:**
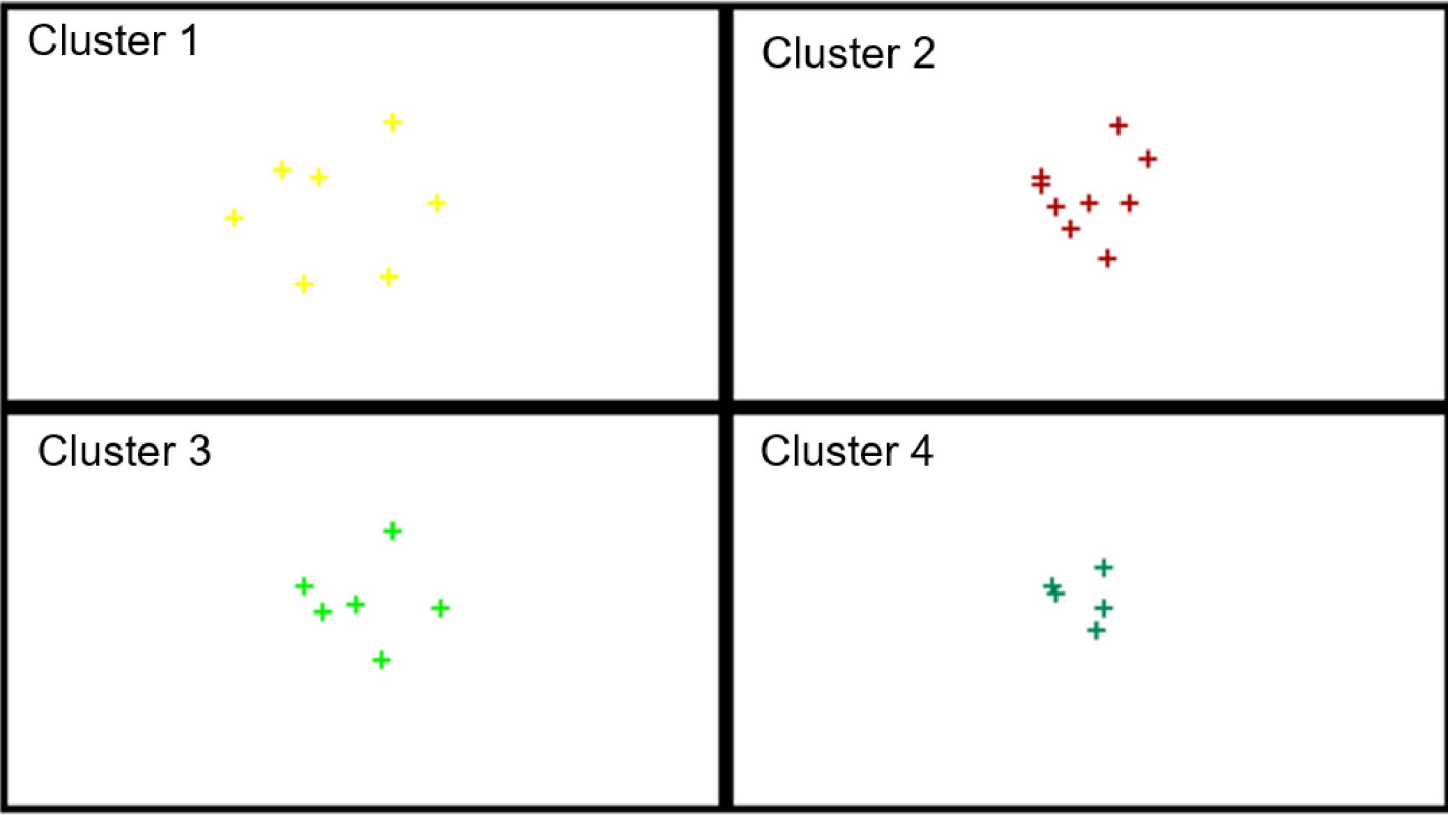
The topological map of similar groups of the EU-27 countries for small enterprises.

**Fig 8:**
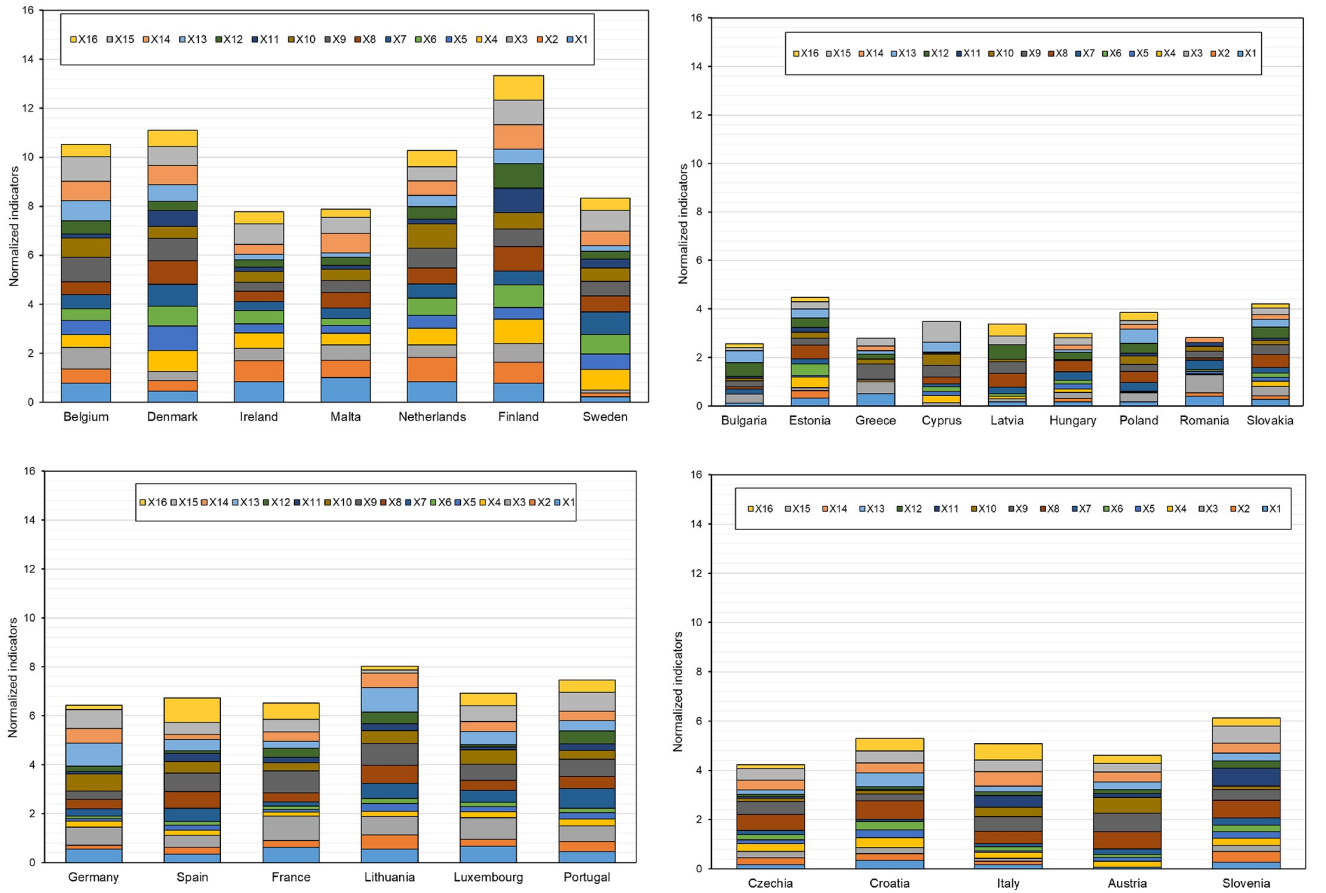
The summary of studied indicators on digitalization and robotization of small enterprises for the EU countries in the designated similar groups (a-cluster 1, b-cluster 2, c-cluster 3, d-cluster 4).

In the next step of the analysis, the EU countries were divided based on the values of indicators for small enterprises only. The determined cluster compositions are presented in Table 8, and their distribution in the created clusters on the basis of activation values is presented on the topological map in Fig 7. In turn, Fig 8 presents the profiles of the indicators adopted for the study.

**Table 8:** The division of the EU countries into similar groups based on the examined indicators for small enterprises together with activation values (distance from the center of the cluster).

Elements of clusters 1	Activation function	Elements of clusters 2	Activation function	Elements of clusters 3	Activation function	Elements of clusters 4	Activation function
Belgium	0.77	Bulgaria	0.56	Germany	0.80	Czechia	0.41
Denmark	0.88	Estonia	0.65	Spain	0.72	Croatia	0.54
Ireland	0.81	Greece	0.68	France	0.66	Italy	0.46
Malta	0.86	Cyprus	0.84	Lithuania	0.83	Austria	0.59
Netherlands	0.65	Latvia	0.66	Luxembourg	0.36	Slovenia	0.60
Finland	1.15	Hungary	0.51	Portugal	0.56		
Sweden	0.96	Poland	0.52				
		Romania	0.79				
		Slovakia	0.30				

Based on the analysis, cluster 1 contains 7 countries, 5 of which have a high level of digitalization and robotization of enterprises, i.e., Belgium, Denmark, Finland, Sweden, the Netherlands, and two at medium-high level, namely Ireland and Malta (based on the TOPSIS analysis). Cluster 2 involves countries with a low level of digitalization and robotization (Bulgaria, Greece, Cyprus, Latvia, Hungary, Poland, Slovakia, Romania) and one country with a medium-low level of digitalization and robotization (Estonia).

**Fig 9:**
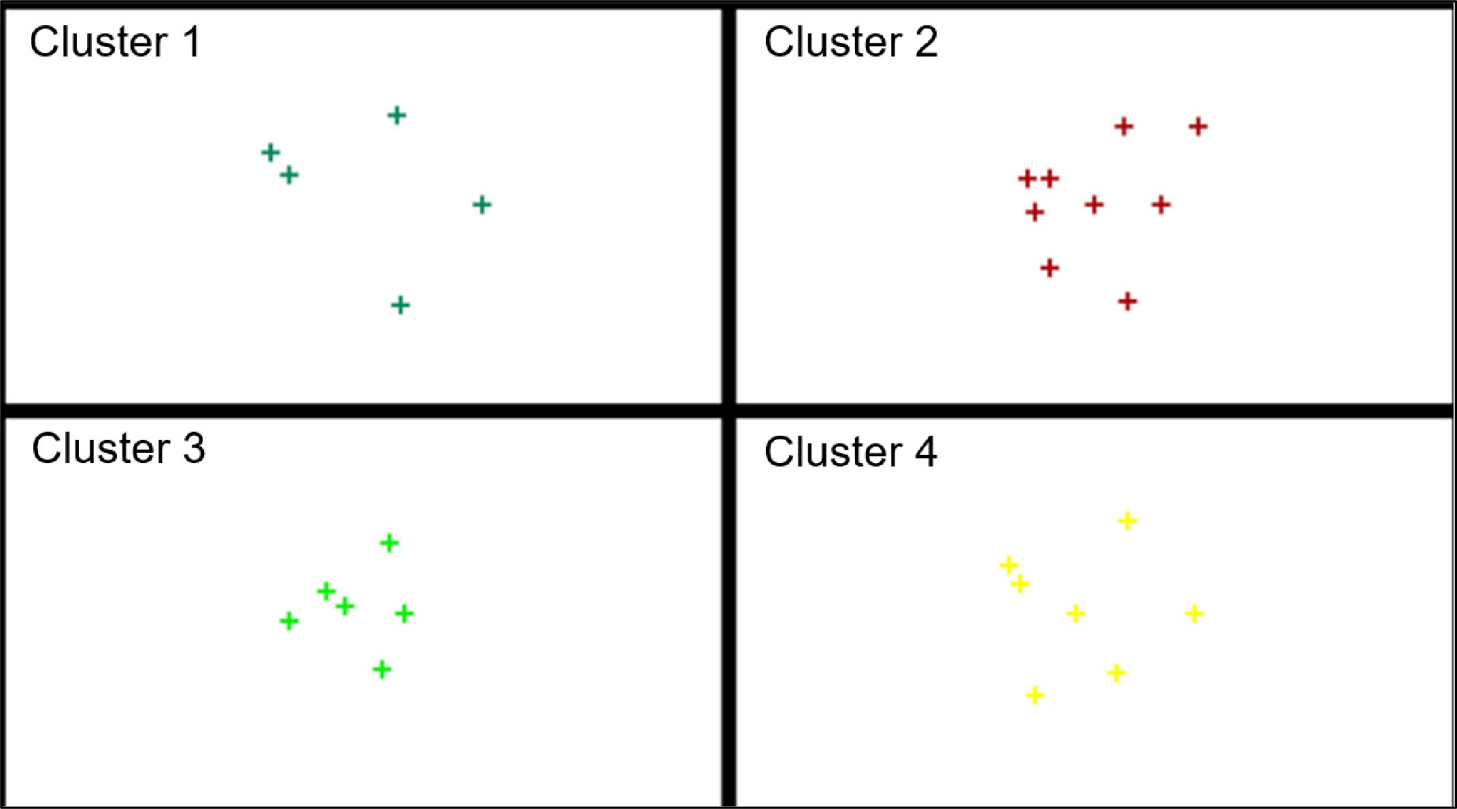
The topological map of similar groups of the EU-27 countries for medium-sized enterprises.

**Fig 10:**
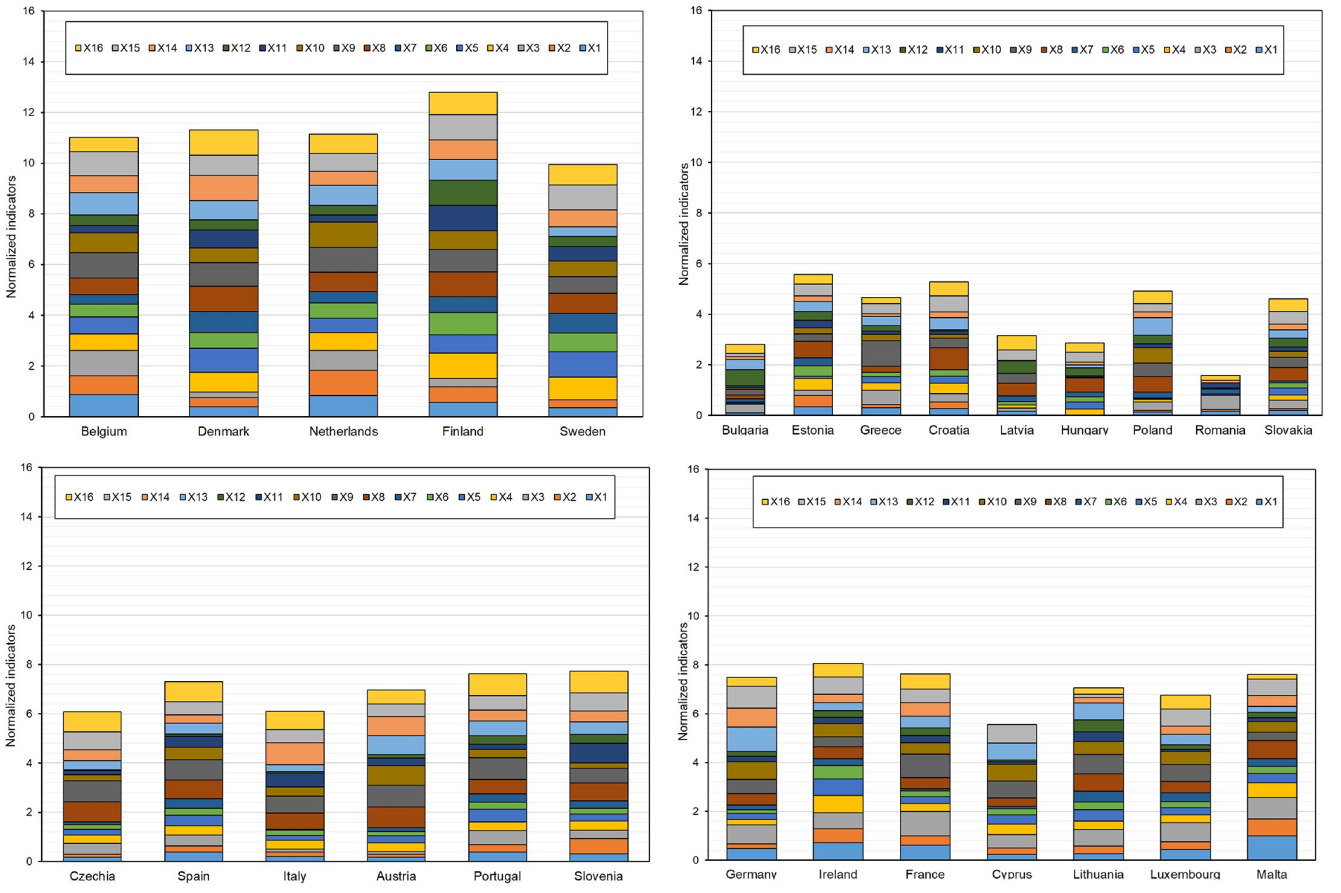
The summary of studied indicators on digitalization and robotization of medium-sized enterprises for EU countries in the designated similar groups (a-cluster 1, b-cluster 2, c-cluster 3, d-cluster 4).

In the next analysis, the EU countries were divided taking into account the digitalization and robotization indicators for medium-sized enterprises. The obtained cluster compositions are presented in Table 9 and their distribution on the topological map in Fig 9. Fig 10 presents the profiles of indicators for countries in individual similar groups.

**Table 9:** The division of the EU countries into similar groups based on the examined indicators for medium-sized enterprises together with activation values (distance from the center of the cluster).

Elements of clusters 1	Activation function	Elements of clusters 2	Activation function	Elements of clusters 3	Activation function	Elements of clusters 4	Activation function
Belgium	0.88	Bulgaria	0.63	Czechia	0.49	Germany	0.78
Denmark	0.68	Estonia	0.66	Spain	0.40	Ireland	0.69
Netherlands	0.81	Greece	0.80	Italy	0.59	France	0.60
Finland	0.76	Croatia	0.71	Austria	0.67	Cyprus	0.78
Sweden	0.82	Latvia	0.54	Portugal	0.51	Lithuania	0.78
		Hungary	0.60	Slovenia	0.66	Luxembourg	0.38
		Poland	0.68			Malta	0.79
		Romania	0.94				
		Slovakia	0.28				

The results showed that Belgium, Denmark, Finland, Sweden and the Netherlands (cluster 1) are the countries with the highest level of digitalization and robotization of medium-sized enterprises. By contrast, Croatia, Poland, Slovakia, Greece, Latvia, Bulgaria, Hungary and Romania from cluster 2 are the countries with the lowest level of this process. When analyzing the profiles of the indicators (Fig 9), it can be concluded that the largest disproportions for medium-sized enterprises are between countries from cluster 2 (the lowest level of digitalization and robotization), and the smallest–between countries from cluster 1 (high level) and cluster 3 (medium level).

**Fig 11:**
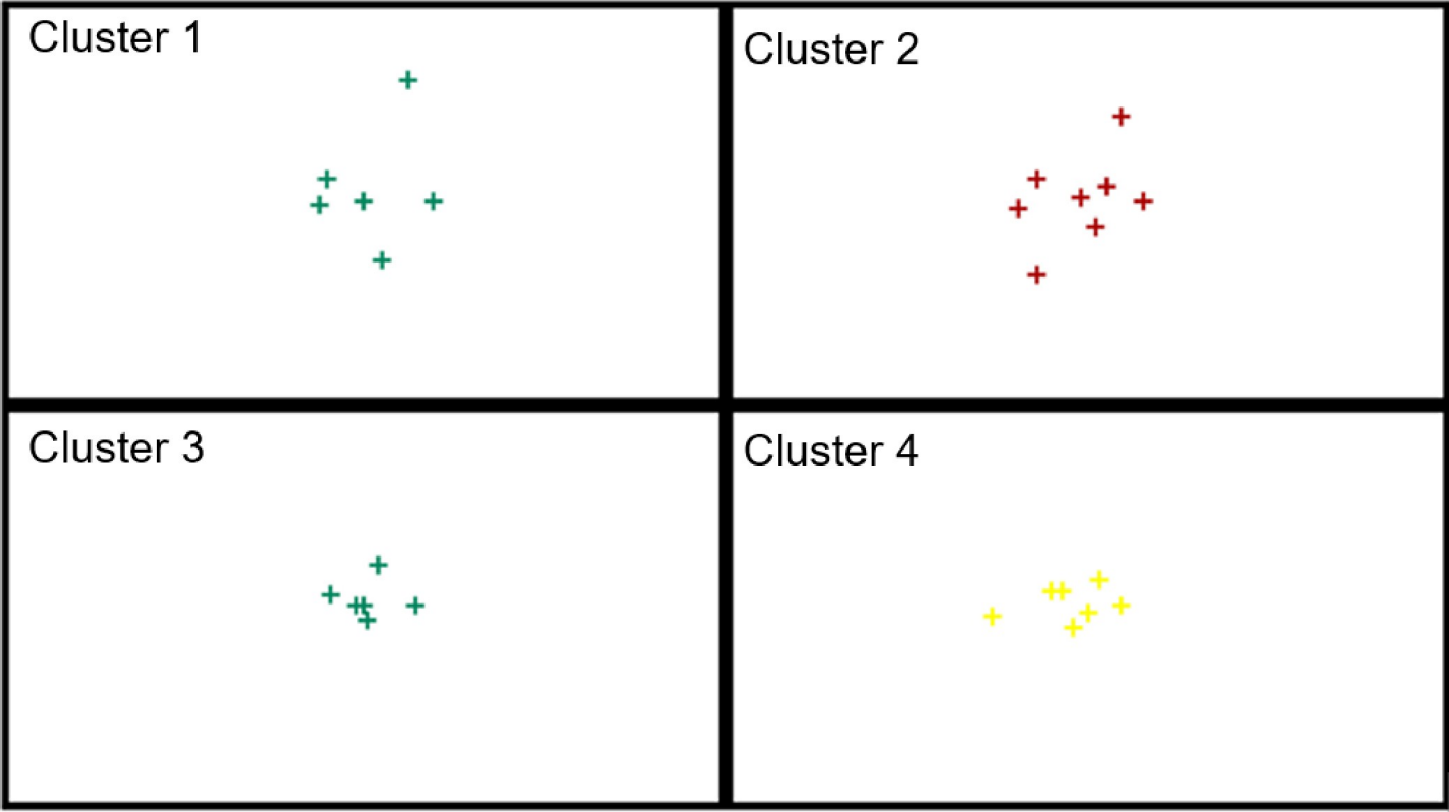
The topological map of similar groups of the EU-27 countries for large enterprises.

**Fig 12:**
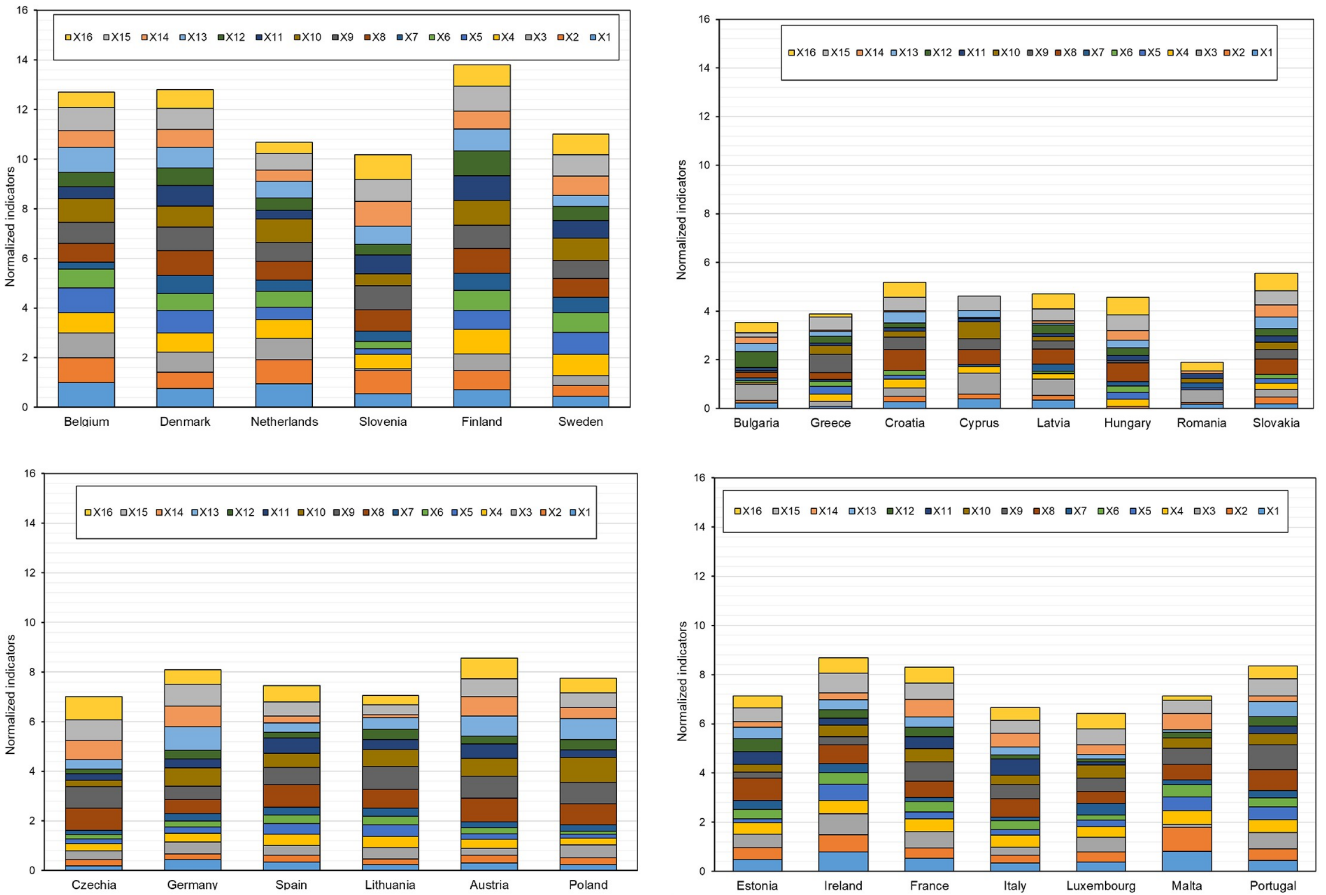
The summary of studied indicators on digitalization and robotization of large enterprises for EU countries in the designated similar groups (a-cluster 1, b-cluster 2, c-cluster 3, d-cluster 4).

The purpose of the next analysis was to determine groups of countries similar in terms of digitalization and robotization of large enterprises. The compositions of the created clusters for these enterprises are presented in Table 10, and their distribution on the topological map is shown in Fig 11. In turn, Fig 12 presents the profiles of digitalization and robotization indicators for countries from each cluster with respect to large enterprises.

**Table 10:** The division of the EU countries into similar groups based on the examined indicators for large enterprises together with activation values (distance from the center of the cluster).

Elements of clusters 1	Activation function	Elements of clusters 2	Activation function	Elements of clusters 3	Activation function	Elements of clusters 4	Activation function
Belgium	0.80	Bulgaria	0.73	Czechia	0.71	Estonia	0.63
Denmark	0.47	Greece	0.74	Germany	0.63	Ireland	0.67
Netherlands	0.76	Croatia	0.59	Spain	0.53	France	0.50
Slovenia	1.10	Cyprus	0.91	Lithuania	0.67	Italy	0.60
Finland	0.66	Latvia	0.51	Austria	0.45	Luxembourg	0.61
Sweden	0.69	Hungary	0.81	Poland	0.50	Malta	0.93
		Romania	0.90			Portugal	0.59
		Slovakia	0.57				

The results indicate that the highest level of digitalization and robotization of large enterprises, were shown by countries from cluster 1 (Belgium, Denmark, the Netherlands, Slovenia, Finland, Sweden), and the lowest–from cluster 2 (Bulgaria, Greece, Croatia, Cyprus, Latvia, Hungary, Romania, Slovakia). The composition of these clusters is the same as in the case of the analysis concerning the total number of enterprises of the EU countries. At the same time, countries in cluster 2 are again characterized by significant variation in the values of digitalization and robotization indicators. Clusters 3 and 4 include countries characterized by significant similarity at the average level of values of these indicators. In their case, the composition changed slightly, Portugal was in cluster 4 (in the case of the analysis variant for all companies, it was in cluster 3), thus showing greater similarity to the countries in this cluster.

## 5. Discussion

The conducted research allowed for the evaluation of the level of digitalization and robotization of enterprises of the EU countries and their similarity in this regard, taking into account the size of production enterprises.

When analyzing the results, it can be concluded that in terms of assessing the level of digitalization and robotization of the EU countries’ enterprises, high levels (for all their groups) were found in Finland, Sweden, Denmark and Belgium and Netherlands. In these countries, the determinants of digitalization and robotization were reported to be at a very high level, exceeding in most cases the average values for the EU27. A high level of digitalization and robotization without taking into account the size of enterprises was also found in Slovenia, and when taking into account the size of these enterprises (small medium-sized and large)–the level was defined as average-high. Sufficient levels of digitalization and robotization of enterprises were also reported in Ireland, France, Austria, Germany and Portugal (in total and for large enterprises). In the case of small enterprises–Germany and France were found to have less favorable results (Fig 4). On the other hand, the countries characterized, in each variant of the analysis, by a low level of digitalization and robotization of enterprises for all their groups were Romania and Greece. For enterprises in total, small and medium-sized, the low levels of digitalization and robotization were also found in Hungary, Bulgaria and Latvia.

In contrast, the countries with low levels of digitalization and robotization of enterprises for all groups of enterprises in each variant of the analysis are Romania and Greece. In the case of total, small and medium-sized enterprises, the low levels of digitalization and robotization were also found in Hungary, Bulgaria and Latvia.

The results of the studies of the assessment of the level of digitalization and robotization of enterprises in the EU countries indicate that more emphasis should be placed on the digitalization of small and medium-sized enterprises, because, as can be seen, large enterprises have much higher rates of this process (Table 6). These results also indicate that the level of digitalization and robotization varies widely across the EU countries. This also applies to individual groups of enterprises. This is an unfavorable phenomenon, as it may significantly hinder the digital transformation process across the EU. In order to avoid such a situation and the further deepening of these differences, there is a need for greater solidarity between countries and assistance provided by the more developed ones to those with a lower level of development.

The aim of the second analysis involved the identification of similar EU countries in terms of assessing the level of digitalization and robotization of enterprises.

All calculations in this regard were carried out on the normalized values of the adopted determinants. The EU countries were divided into four similar groups. This division was made for the whole economy, also taking into account the size of enterprises. The results showed that the similarity of countries, broken down into these four variants differed. The level of digitalization of the whole economy is not always evenly distributed across all enterprises. This is due to the different structure and capabilities of these companies and the incentive policies applied by countries. For each variant, countries with the highest degree of digitalization and robotization were found to be in cluster 1 while those with the lowest degree of digitalization in cluster 4.

The clear leader in the EU-27 countries in terms of the levels of digitalization and robotization of all types of enterprises is Finland. It is one of the most digitized countries in the world [76]. Finnish companies are very keen to use digital technologies, in particular for cloud computing services and electronic invoices. The same is true in Sweden, where companies are using digital technologies to improve efficiency, productivity and sales. Businesses in this country are keen to use cloud computing services and buy high CC services (accounting applications, CRM software, computing power).

Denmark is another leader from Scandinavia in the use of digital technologies by business. More and more companies are using cloud computing services and electronic invoices. Denmark provides access to high-speed broadband Internet, and the take-up of high-speed Internet connections is growing steadily and is well above the EU-27 average [70]. Danish companies are also characterized by a high ability and willingness to use new technologies and introduce changes in the work system. As high as 50% of enterprises there use ERP systems, which after Belgium is the second result in the EU. Sweden and Denmark were ranked as the 5th and 6th most robotic (automated) countries in the world in 2019, respectively [80]. In Sweden, the number of robots per 10,000 employees was found to amount to 274 units and in Denmark to 243 units.

Among Western countries, only Belgium and the Netherlands achieved a comparable high level of digitalization and robotization of enterprises. In Belgium, the number of robots per 10,000 employees was found to equal 214 units, and in the Netherlands– 194 units [80]. The high rating is due to, among other things, digital maturity in the use of big data technologies [53] or the use of cloud computing services. The degree of robotization of enterprises in these countries exceed the EU average.

It is therefore clear that these countries, being the leaders of digitalization in Europe, should share their experiences with other countries to a greater extent.

The main task for the EU-27 leaders in terms of digitalization and robotization of enterprises is to further increase this level and help other countries to achieve such results. The division into similar groups is also intended to identify groups of countries for broader cooperation and exchange of experience and assistance. Mutual assistance and exchange of experience is the basis of the EU common market.

It can be observed that the lowest results were achieved by countries of Central, Eastern and South-Eastern Europe. Despite the dynamic economic development of these regions in terms of digitalization and robotization, the situation is not satisfactory [79,81]. Besides, the overall economic level of these regions needs significant improvement.

In order for the economies of these countries to reach an appropriate level of development, it is necessary to base them on knowledge and modern technologies, as well as the experience of the most developed countries. In this regard, a great opportunity is created by the digitalization, automation and robotization of production processes [82]. The changes should include all groups of companies in these regions, because currently the level of their digitalization and robotization is low. This concerns mainly the automation and robotization of production, the use of 3D printing, big data analysis and cloud computing [58,83–85]. There are also problems in access to broadband Internet, which is related to the later adoption of digitalization activities. It can be noted that among the countries, characterized by a low level of digitalization and robotization of enterprises in each variant of the analysis, only Greece belongs to the so-called "Old" EU countries. The rest are countries of the "New Union".

The exception in this regard is the Czech Republic, which, when compared to the rest of the countries of Central, Eastern and Southeastern Europe, is distinguished by high innovation, competitiveness and a relatively high level of digital transformation of manufacturing and service and commercial enterprises [82,86,87].

Thus, the results obtained indicate a large variation in the level of digitalization and robotization of manufacturing enterprises in the EU27 countries, which are also related to their geographical location and the time of accession to the EU.

In terms of the assessment of the level of digitalization and robotization, the best and the weakest groups of countries, to a large extent, coincide with the results of The Digital Economy and Society Index (DESI). The differences that occur are related to other indicators adopted for the analysis and the fact that in this study the research was carried out for companies and not entire societies [79].

To sum up, the research and the results show that the level of digitalization and robotization of the EU countries varies greatly. The analysis of this level in enterprises of different sizes further deepens these differences. Also, large differences in this level for different groups of enterprises within a given country were oftentimes noted. The greatest discrepancies were found for the group of small and medium-sized enterprises, which clearly indicates the direction in which the digitalization policy should go.

It should also be emphasized that despite the application of two independent methods of analysis, the results were reported to be similar. It is interesting in that the objectives of these analyses were different. The analysis based on the TOPSIS method was to determine the order (classification) of the EU countries in terms of the level of digitalization and robotization. On the other hand, the division into similar groups was to indicate the groups of the EU countries in which individual indicators showed the greatest similarity. This in turn should translate into potential opportunities for cooperation and building digital coalitions, for example, when applying for EU funds.

**Fig 13:**
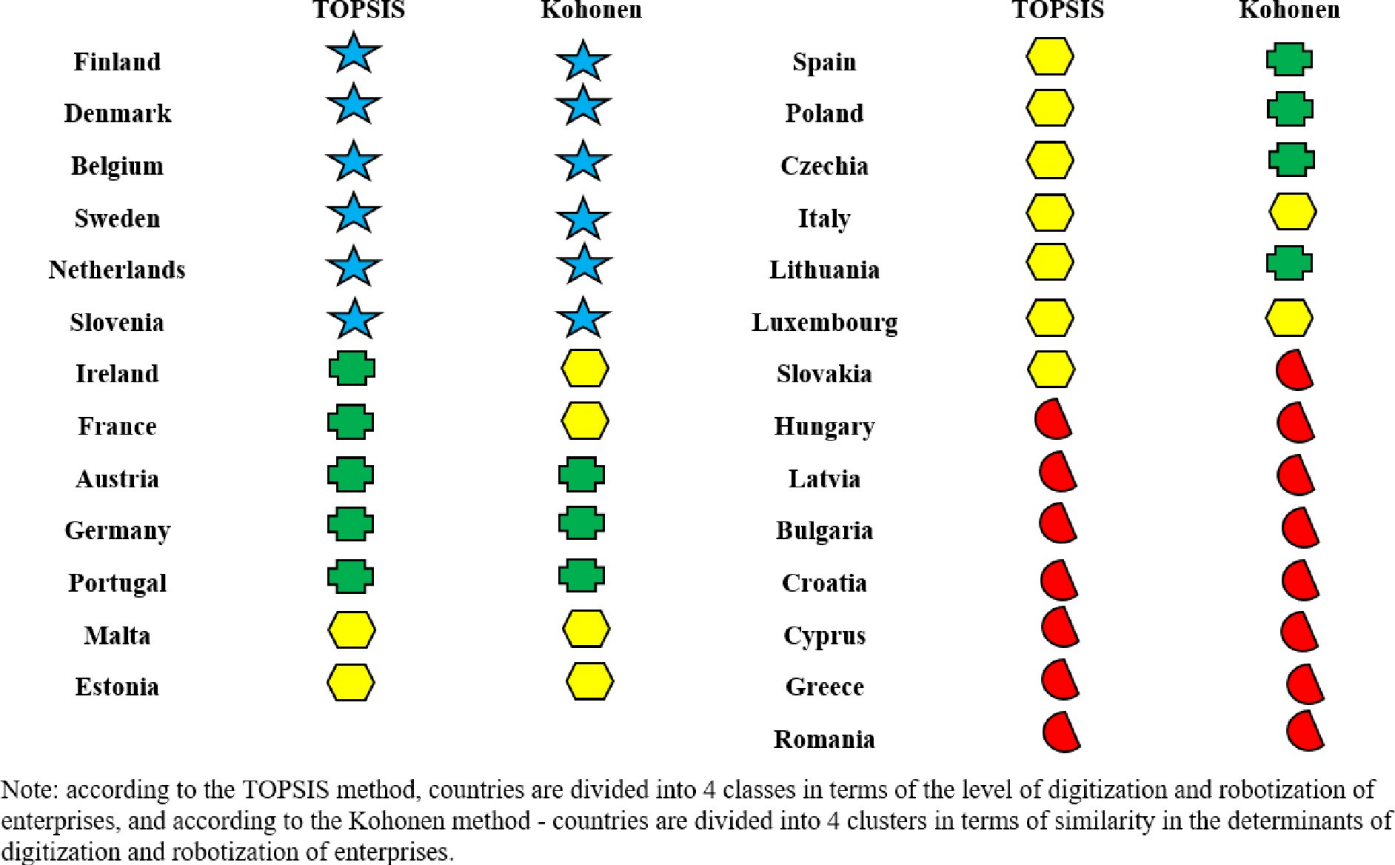
The summary of the results of the study of the level of digitalization and robotization of the EU countries and the similarity between these countries.

Despite the different objectives, the results of both analyses provide a lot of information about the advancement of digitalization and robotization processes in the EU countries. The combination of the results of these two analyses, taking into account all enterprises of individual countries is presented in Fig 13.

The results in both analyses provide great opportunities for interpretation, inference and formulation of recommendations for future activities related to the process of digitalization and robotization in the EU countries. At the same time, they provide answers to all three formulated research questions.

## 6. Limitations and directions for further research

The methodology developed, the research conducted, and the results obtained allowed the authors to formulate an opinion on the limitations of this methodology and future research directions.

In terms of limitations that may have affected the results of the study, the timeliness of data used in the study should be mentioned. It should be emphasized that this study used the latest available data from 2018 on the digitalization and robotization of enterprises.

However, as for the directions of further research, it is worth considering the increase in the number of indicators adopted for the analysis, also representing possible new areas of digitalization and robotization development. On the other hand, in order to assess the changes, in this area in subsequent years, and compare them with the results presented in this paper, it is reasonable to carry out a study in accordance with the principles adopted in this work. Besides, it would be extremely interesting, with the acquisition of further data, to complement this analysis with further results and on this basis to observe changes in individual countries.

In terms of the adopted indicators, a more detailed analysis for individual countries would also be extremely interesting. This paper omits this analysis, as it would have greatly increased its already large content. However, a more detailed analysis of changes in individual indicators, for the studied groups of enterprises in individual countries would allow a very precise diagnosis of the state of digitalization and robotization.

Research comparing the level of digitalization and robotization of various economic sectors (according to NACE Rev. 2) in these countries would also bring valuable information. By extending the research with the results of the analysis of the level of digitalization and automation in selected sectors of the economy (e.g., for transport, energy, agro-food industry, machinery industry, health care, and others), it would be possible to perform an even more precise analysis of the state of advancement of these processes.

It should also be stressed that the developed methodology has a universal character and can be successfully used to study other groups of countries as well. The results of such research in comparison with the results for the EU countries would be very interesting material on the level of digitalization, both from the scientific and utilitarian point of view.

## 7. Conclusions and policy implications

The analysis of the state of the world economy in recent years clearly shows that the Internet and digital technologies have significantly changed this economy. In practically all areas of economic, social and political life, changes associated with the dynamically developing idea of Industry 4.0 are visible. We are witnessing the formation of a digital economy, which has an increasing impact on the labor market, consumption, the functioning of state and local government institutions, as well as on political life. In addition to many advantages, this economy also brings a number of risks, some of which were previously unknown. Changing employment structure, digital exclusion, digital surveillance, cyber threats and many others are growing problems that may slow down the pace of change. What is clear, however, is that change will happen, and probably even faster than expected. The advantages of the process of digitalization of the global economy are disproportionate to the emerging threats, and at this stage there is no turning back from this process. The SARS-CoV-2 coronavirus pandemic makes this even more apparent [88–90]. Also, the fascination with the digital world by younger generations clearly indicates the direction in which the global economy is heading [91].

To meet the considerable demands of this process, individual countries and groups of countries (such as the EU-27) are even forced to implement digital transformation. However, this process requires large financial resources as well as organizational and social changes. Individual countries, especially smaller and less wealthy ones, may find it increasingly difficult to keep up with the implementation of these changes. Therefore, it becomes necessary to build different types of "digital coalitions" between countries and companies in order to compete and improve the quality and safety of their citizens’ lives.

The analysis of the level of digitalization and robotization of enterprises of the EU countries and the identification of similarity of these countries, taking into account selected indicators, presented in the paper, is part of this issue. Of fundamental importance for these activities was the determination of indicators (determinants), which characterize the most important areas of the digitalization and robotization process. On the other hand, the main objectives were to delineate the ranking of EU countries in terms of the level of digitalization and robotization taking into account the size of enterprises and to determine similar groups of these countries in this regard.

The results confirm the diversity of the EU countries and indicate the large differences in the process of digitalization and robotization in these countries that exist between countries and between different groups of companies in each country.

These results clearly show the great challenges that the EU authorities need to face to reduce these differences and so increase the pace of digital transformation to meet global competition. This is considered crucial since the EU can take action related to digital transformation within the framework of sectoral and horizontal programs and on the basis of the provisions of the Treaty on the Functioning of the European Union. These provisions are binding for the Member States, which have to implement them. In addition to the general guidelines, these activities also include the financing of various types of programs including the digitalization and robotization of enterprises. Undoubtedly, the centralization of funding for digital transformation offers great opportunities for success. Despite some risks, it makes it possible to target the least developed areas and subsidize them accordingly. However, this targeting must be preceded by a very thorough analysis of these areas and the legitimacy of their funding and clearly defined objectives.

The results presented in the paper can be used to improve the process of financing digitalization and robotization activities of the EU countries. In addition to ranking countries in terms of the level of digitalization and robotization, they also indicate the groups of similar countries, taking into account the size of enterprises.

When considering the overall degree of digitization (for all enterprises), similar groups with the highest (1) and lowest (4) degree of digitization have the following compositions:

- Cluster 1: Belgium, Denmark, the Netherlands, Slovenia, Finland, Sweden;
- Cluster 2: Bulgaria, Greece, Croatia, Cyprus, Latvia, Hungary, Romania, Slovakia.

When taking into account small enterprises, the composition of these groups is as follows:

- Cluster 1: Belgium, Denmark, Ireland, Malta, the Netherlands, Finland, Sweden;
- Cluster 2: Bulgaria, Estonia, Greece, Cyprus, Latvia, Hungary, Poland, Romania, Slovakia.

For medium-sized enterprises, the group compositions are as follows:

- Cluster 1: Belgium, Denmark, the Netherlands, Finland, Sweden;
- Cluster 2: Bulgaria, Estonia, Greece, Croatia, Latvia, Hungary, Poland, Romania, Slovakia.

However, for large enterprises the composition is as follows:

- Cluster 1: Belgium, Denmark, the Netherlands, Slovenia, Finland, Sweden;
- Cluster 2: Bulgaria, Greece, Croatia, Cyprus, Latvia, Hungary, Romania, Slovakia.

This information should mobilize countries to strengthen cooperation and jointly apply for EU funds, also for specific groups of enterprises. This cooperation should therefore include not only countries but also groups of enterprises from different countries. Joint investments in key areas related to digital transformation (included in the examined determinants) create great opportunities for success and use of these solutions by all EU countries [92]. Especially in the case of small and medium-sized enterprises [93] and less wealthy countries, such a way of development seems to be most reasonable. On the other hand, the leaders of this transformation should support less developed countries in this area, both financially and in terms of organization and science. Building a common European economy requires broad cooperation and an attitude of solidarity among all its members.

It is also extremely important to take action to eliminate not only the differences within the Union as a whole, but also the differences between business groups in individual Member States. In this regard, particular attention should be paid to the countries of Central, Eastern and South-Eastern Europe, which require significant assistance in this area. The continuation of such disparities between the EU countries in terms of digitalization and robotization may lead to an increase in social and economic inequalities and digital exclusion between these countries.

Good practices and mutual assistance between countries should also foster the better use of transmission capacity, the development of innovative solutions, a knowledge-based economy as well as the research and development of new digital technologies. The main instrument for realizing the vision of modern, competitive and industrialized Europe should be a coherent and resilient single European market, which has been introduced to the digital age for several years now. In order to survive as a community, the EU must meet the challenges that have emerged as a result of the "diffusion" of advanced digital technologies in the global economy, especially when considering the digitalization of the economy, which is now being promoted as a pillar of the EU’s economic recovery from the SARSCovid-19 pandemic.
